# CAR T-cells for the treatment of CNS malignancies

**DOI:** 10.3389/fimmu.2026.1891659

**Published:** 2026-09-03

**Authors:** Caleb A. Kisamore, Paul J. Owolabi, Claire O. Kisamore, William H. Walker

**Affiliations:** 1 Department of Neuroscience, Rockefeller Neuroscience Institute, West Virginia University Cancer Institute, West Virginia University, Morgantown, WV, United States; 2 Department of Medical Oncology, West Virginia University Cancer Institute, West Virginia University, Morgantown, WV, United States

**Keywords:** adoptive cell transfer, brain metastases, brain tumors, cancer immunotherapy, CAR T-cells

## Abstract

Chimeric antigen receptor (CAR) T-cell therapy has undergone great advances since the development of the first-generation CAR in the early 1990s. Currently there are seven FDA approved CAR T-cell therapies for the treatment of hematological malignancies, with marked clinical success. However, the application of CAR T-cell therapies to solid tumors, particularly CNS malignancies, provide unique challenges. This review provides historical perspective on CAR T-cell therapy, describes CAR structures and current generations, and details challenges with the application of CAR T-cells to CNS malignancies. In addition, an extensive summary of all clinical trials, as of March 2026, utilizing CAR T-cell therapy for primary and secondary CNS malignancies are provided. Specific attention is given to detailing CAR structure, route of administration, and dosing. Furthermore, outcomes and adverse events for each trial are described for completed studies and studies reporting interim results. As the application of CAR T-cell therapy continues to expand in CNS malignancies, it is imperative that clinical studies provide adequate details on CAR structures, try to more quickly adopt/act on preclinical successes, and seek novel application strategies to advance growth in the field.

## 1 Introduction

Chimeric Antigen Receptor (CAR) T-cell therapy (i.e., the adoptive transfer of patient-derived T lymphocytes genetically engineered to target and destroy cancer cells) represents a revolutionary paradigm shift in the treatment of cancer. The earliest iteration of the modern CAR can be traced back to 1987 when Japanese immunologist Dr. Yoshikazu Kurosawa and colleagues demonstrated that a chimeric receptor derived from the variable region of an anti-phosphorylcholine (PC) antibody and ligated to the constant region of a murine T-cell receptor (TCR) possessed the capacity to activate an immune response when exposed to PC antigen (1, 2). Two years later, in 1989, Israeli immunologist Dr. Zelig Eshhar and colleagues sought to construct a non-MHC restricted T-cell with antibody-like specificity via recombination of the immunoglobulin (Ig) V_H_ and V_L_ to the C-region of TCR α and β chains, resulting in a double-chain heterodimeric chimeric TCR (cTcR) capable of trafficking to and killing target cells (2, 3). Later, in 1993, Eshhar updated the previous construct which dramatically improved the transduction efficiency and antitumor efficacy while formally establishing the first-generation CAR T-cell (2, 4). Since then, multiple successive modifications to the first-generation CAR construct have led to improved safety and efficacy profiles and the emergence of five generations of CARs.

CAR T-cells have achieved remarkable clinical success in the context of hematological malignancies. For example, several multicenter trials have reported that complete response (5, 6) to CD19 CAR T-cell therapy for relapsed and/or refractory (R/R) B-cell malignancies was observed in 40-54% of R/R B-cell lymphomas (7–9), 67% of R/R mantle cell lymphomas (10), 69-74% of indolent B-cell lymphomas (11, 12), and 71-81% of R/R B-cell acute lymphoblastic leukemia (13, 14). Such tremendous success has resulted in the FDA approval of seven CAR T-cell therapies, as of this publication (**Table 1****) (**15, 16**).**

**Table 1:** The 7 current FDA approved CAR T-cell therapies.

Name	Construct (scFv-HR-TMD-CD-ICSD)	Disease	Year approved
Kymriah^®^	anti-CD19-CD8α-CD8α-4-1BB(CD137)-CD3ζ	r/r B-ALL	2017
		r/r LBCL after ≥2 lines of ST	2018
		r/r FL after ≥2 lines of ST	2022
Yescarta^®^	anti-CD19-CD28-CD28-CD28- CD3ζ	r/r LBCL after ≥2 lines of ST	2017
		r/r FL after ≥2 lines of ST	2021
Tecartus^®^	anti-CD19-CD28-CD28-CD28- CD3ζ	r/r MCL	2020
		r/r B-ALL	2021
Breyanzi^®^	anti-CD19-IgG4-CD28-4-1BB(CD137)-CD3ζ	r/r LBCL after ≥2 lines of ST	2021
		r/r LBCL and not eligible for HSCT	2022
		r/r CLL or SLL after ≥2 lines of ST	2024
Abecma^®^	anti-BCMA-CD8α-CD8α-4-1BB(CD137)-CD3ζ	r/r MM after ≥4 prior lines of therapy	2021
Carvykti^®^	anti-BCMA-CD8α-CD8α-4-1BB(CD137)-CD3ζ	r/r MM after ≥4 prior lines of therapy	2022
Aucatzyl^®^	anti-CD19-CD8α-CD8α-4-1BB(CD137)-CD3ζ	r/r B-ALL	2024

anti-BCMA, anti-B-cell maturation antigen; scFv, single chain variable fragment; R/R, relapsed or refractory; B-ALL, B-cell precursor acute lymphoblastic leukemia; LBCL, large B-cell lymphoma; FL, follicular lymphoma; MCL, mantle cell lymphoma; ST, systemic therapy; HSCT, hematopoietic stem cell transplantation; CLL, Chronic lymphocytic leukemia; SLL, small lymphocytic lymphoma; MM, multiple myeloma.

Despite this success, studies have reported several serious toxicities associated with CAR T-cell therapy, such as: cytokine release syndrome (CRS) (15, 16), immune effector cell associated neurotoxicity syndrome (ICANS) (17, 18), tumor inflammation-associated neurotoxicity (TIAN) (19), and anaphylaxis (20). Furthermore, the utility of CAR T-cells in treating solid tumors, including CNS malignancies, has failed to mirror the success seen in hematological malignancies. For example, a meta-analysis conducted in 2019 encompassing 22 studies reporting on neurologic tumors, hepatobiliary and pancreatic cancers, melanoma and sarcoma, gastrointestinal malignancies, prostate cancer, breast cancer, non-small-cell lung cancer, and others (n=262) found that the overall pooled response rate was only 9% (21). Multiple hypotheses have been presented to account for this disparity, including antigen heterogeneity, the immunosuppressive tumor microenvironment, and difficulty trafficking into the tumor (22–27). Current research efforts are being directed toward optimizing the therapeutic strategies of CAR T-cells to increase their efficacy across different cancers and minimize associated toxicities (28). Here, we review existing literature on CAR T-cells to provide an overview of this evolving immunotherapy and to offer insights on how to expand the frontiers of its application in CNS malignancies.

## 2 Overview of the CAR structure

**Figure 1:**
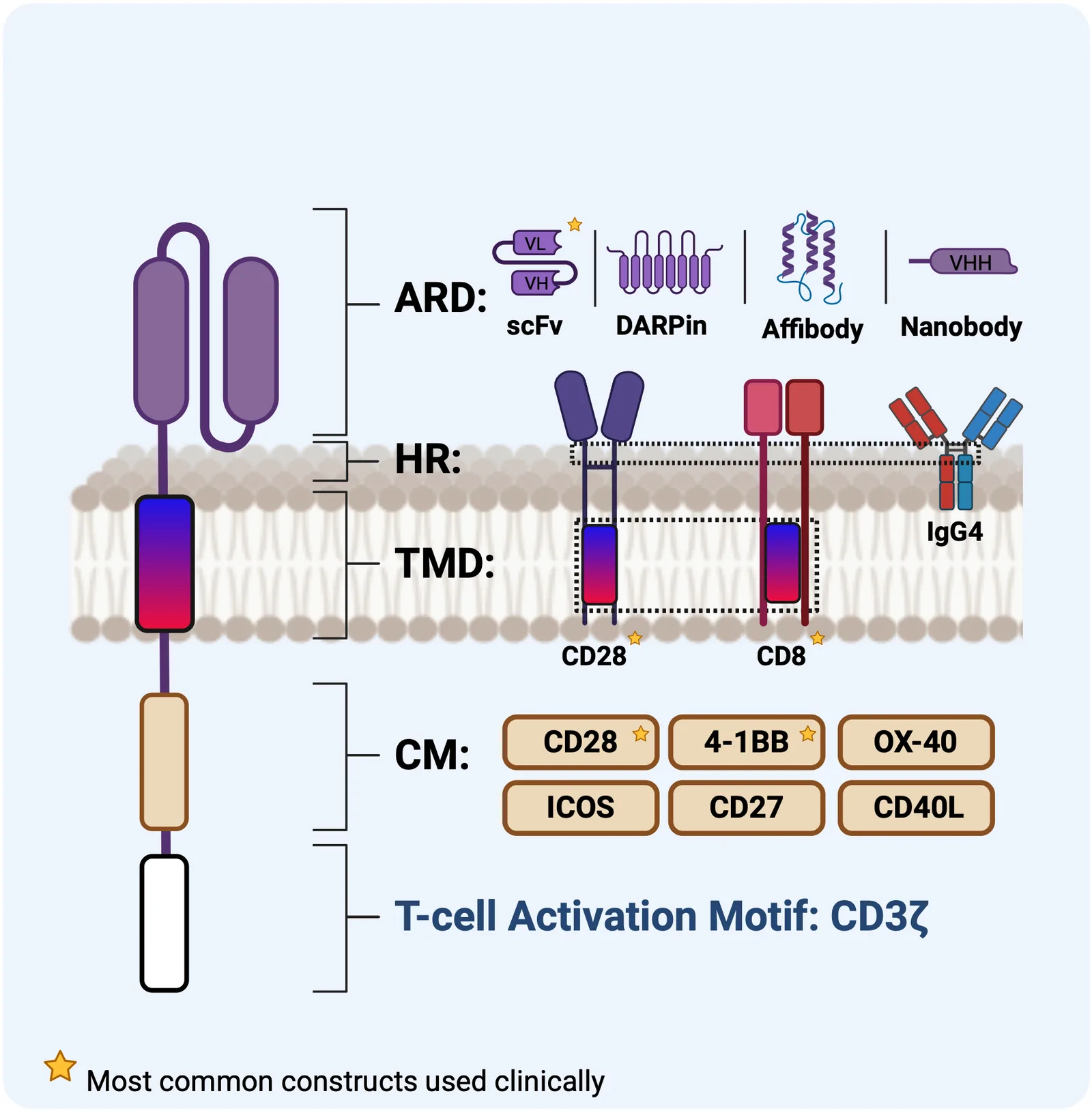
Schematic of chimeric antigen receptor (CAR) construct and molecular signaling pathways of FDA approved CARs. Five functional domains ligated in series make up the molecular construct of all FDA approved CARs. These domains include the antigen recognition domain (ARD), Hinge Region (HR), Transmembrane Domain (TMD), costimulatory molecule (CM) domain, and the primary signaling domain (CD3ζ). A variety of molecular constructs (e.g., scFv, DARPin, Affibody, Nanobody as the ARD) can be used for each respective domain, enabling a wide range of possibilities. Created with biorender.com.

In the following section we will briefly mention the three fundamental CAR domains and consider how successive generations have evolved through iterative modifications to the endodomain (**Figure 1**).

### 2.1 The ectodomain

The ectodomain is the outermost structure of the CAR and consists of an antigen recognition domain (ARD) and hinge region (HR). The most common ARD is an antibody-derived single-chain variable fragment (scFv) which consists of a heavy (V_H_) and light (V_L_) chain variable region, respectively, connected by a flexible linker. One reason for its popularity includes the ability to modulate the affinity and avidity of the scFv via stereochemical modifications of critical ligand-binding regions. This allows researchers to fine-tune CAR T-cell effector functions such as cytokine secretion, proliferation, and persistence while avoiding “on target, off tumor” toxicity, CRS, and ICANS (29–34). Furthermore, scFv-based CAR T-cells offer the added advantage of targeting non-peptide antigens and allowing CAR T-cells to circumvent the immune evasion mechanism of MHC downregulation through MHC-independent antigen recognition (35, 36). Though less common, the ARD can include non-antibody derived structures, such as engineered binding scaffolds (DARPins, affibodies, nanobodies, and monobodies) or natural ligands and receptors (Natural Killer group 2 member D [NKG2D], lymphocyte function-associated antigen-1 [LFA-1] and the TCR variable region [TCR_V_]) (37, 38).

The HR links the ARD to the transmembrane domain (TMD) (39), and is largely responsible for determining the CAR’s membrane transfer efficiency rate, antigen-specific cytotoxicity, and delivery of perforin and granzyme across the immune synapse due to the supramolecular interactions with surrounding membrane proteins and unique stereochemical geometry. Such properties stem from the tertiary protein structure of the HR which directly impacts the thermodynamic stability, signaling threshold, and epitope-paratope interactions of the CAR (40–42). The most commonly employed HRs derive from the extracellular regions of CD8, CD28, and IgG4 (with or without the CH2-CH3 motif of the Fc region), each of which confer distinct physical and chemical properties to the CAR, enabling flexible, optimizable CAR construct design (24, 41, 43, 44). Careful consideration of the selected HR is essential to ensuring optimal performance of the CAR T-cell. For example, several studies have demonstrated that achieving macromolecular complementarity between HR length and the membrane proximity of tumor epitopes (i.e., employing a long HR for membrane-proximal epitopes) results in improved activation, proliferation, and anti-tumor efficacy of the CAR T-cell (43, 45–48). In addition to modulating effector functions of CAR T-cells, the HR can be engineered to express epitopes that are specifically targeted by antibodies or other CAR T-cells to improve elimination *in vivo (*49, 50*)*. Applying the same principle of HR modification to the manufacturing landscape can lead to improved *ex vivo* isolation and enrichment protocols and even *in vivo* CAR T-cell phenotyping from clinical samples (50–52).

### 2.2 The transmembrane domain

The transmembrane domain (TMD) anchors the ectodomain to the endodomain, supporting the stability, expression, and cellular localization of the CAR (41). The most common TMDs used in CAR constructs are derived from the endogenous transmembrane glycoproteins CD8 and CD28, which are constitutively expressed on cytotoxic and naïve T-cells, respectively (53, 54). Selection of the TMD most often depends on the identity of the adjacent extracellular HR or intracellular signal transduction domain, as evidence demonstrates CAR constructs with HR/TMD or TMD/Signal Transduction Domain derived from the same molecule exhibit superior anti-tumor efficacy (41, 55). The structural motif of the HR/TMD has also been shown to significantly affect the activation threshold and immune synapse formation of CAR T-cells independent of the ARD and signal transduction domain (56). Recently, investigators studying the immunological phenomenon known as protein trogocytosis (i.e., the horizontal transfer of membrane-associated molecules, including CARs, between immune cells) have discovered the process is TMD-dependent (57), and therefore suggest that alteration of the TMD is a possible anti-tumor optimization method that needs to be explored further.

### 2.3 The endodomain

The endodomain, also commonly referred to as the intracellular signal transduction domain, is involved in the transduction and amplification of signals required to activate CAR T-cells following antigen recognition (58). Incremental alterations to endodomain architecture has driven the evolution of five CAR generations (59), therefore our discussion of this domain will mirror the timeline from the first generation onward.

#### 2.3.1 The first-generation CAR

The first generation CAR, developed by Eshhar in 1993 (3), was constructed by ligating an antibody-derived scFv to CD3ζ (CD247), a physiological T-cell signaling motif involved in the formation of native TCR/CD3 complexes (60, 61). CD3ζ was selected in part due to the presence of three immunoreceptor tyrosine-based activation motif (ITAM) sequences which are phosphorylated upon receptor-ligand interaction, revealing the binding sites for the kinase ZAP70 resulting in a cascade of downstream signaling via mitogen-activated protein kinase (MAPK), nuclear factor-kappa B (NF-kB), and nuclear factor of the activated T-cell (NFAT) signaling pathways (62, 63). Despite promising *in vitro* performance, the lack of co-stimulation rendered first generation CARs obsolete as anergic responses rapidly exhausted the cells *in vivo* (64).

#### 2.3.2 Second generation CARs

Following dismal *in vivo* activity of first generation CAR T-cells, Sadelain and colleagues engineered the second generation CAR in 1998 by introducing the costimulatory molecule (CM) CD28 in the endodomain (65). Currently, all seven FDA approved CAR T-cell therapies utilize the second generation construct with CD28 or 4-1BB as the CM. Head-to-head comparisons of CD28 and 4-1BB CMs have produced mixed or even conflicting findings resulting in extensive debate regarding which produces “superior” anti-tumor activity. Despite this controversy, it is generally agreed upon that CD28 co-stimulation leads to acute, robust anti-tumor activity with tonic-signaling resulting in significant early cytokine production, early CAR T-cell exhaustion, and differentiation into effector-memory cell phenotypes while 4-1BB co-stimulation results in a delayed but prolonged cytokine response and increased persistence that favors differentiation into a central-memory phenotype (55, 65, 66). Additional CMs that have been employed include ICOS (inducible T cell co-stimulators), CD27, OX-40 (CD134) and CD40L (67). A tremendous amount of research has been dedicated to evaluating the impact of various CMs on CAR T-cell function in the context of hematological and solid tumors, the details of which goes beyond the scope of this review but can be found elsewhere (55, 60, 66, 68, 69).

#### 2.3.3 Third generation CARs

Third generation CARs emerged in the early 2010s as researchers were eager to build upon the success of second generation CARs through the addition of a second CM (70). It is hypothesized that the desired qualities of both CD28 and 4-1BB might be maximized while the undesirable qualities minimized, such that third generation CARs would produce robust and prolonged responses that do not result in early exhaustion or delayed killing via the action of CD28 and 4-1BB co-stimulation, respectively (71, 72). Many studies investigating third generation CARs failed to generate consistent results, with some reporting superiority to second generation CARs (72, 73) and others reporting no difference or even a reduction in function (74–76). Currently, no third generation CAR T-cells have been approved by the FDA.

#### 2.3.4 Fourth generation CARs

Fourth generation CAR T-cells, also known as “armored CARs” or T-cells redirected toward universal cytokine killing (TRUCK), were engineered to circumvent the immunosuppressive TME characteristic of solid tumors via constitutive or inducible (i.e., upon antigen recognition) expression of transgenic proteins, namely immunomodulatory cytokines that enhance CAR T-cell survival and anti-tumor activity (77–79). The modified structure often consists of a second-generation construct backbone with the addition of an inducible expression cassette driven by (NFAT)_n_ response-elements and the IL-2 minimal promoter. Preclinical models exploring the conditional expression of several soluble signaling molecules, such as common gamma chain (γ_c_) cytokines (i.e., IL2 (80, 81), IL-7 (79, 82), IL-15 (83–86), IL-21 (87), and IL-23 (88, 89)) or synthetic orthogonal cytokine receptors (i.e., IL-4R/IL-2R (90, 91), GM-CSF/IL-18R (92), IL-7Rα/IL-2Rβ (93), etc.), have demonstrated the capacity to orchestrate an organized and local immune response via autocrine and paracrine signaling. Of note, these conditional immune responses have been capable of overcoming the immunosuppressive milieu of the TME while mitigating systemic cytokine related toxicity. Currently, several clinical trials utilizing fourth generation CARs are underway and represent a promising frontier in CAR T-cell cancer immunotherapy (94).

#### 2.3.5 Fifth “next generation” CARs

**Figure 2:**
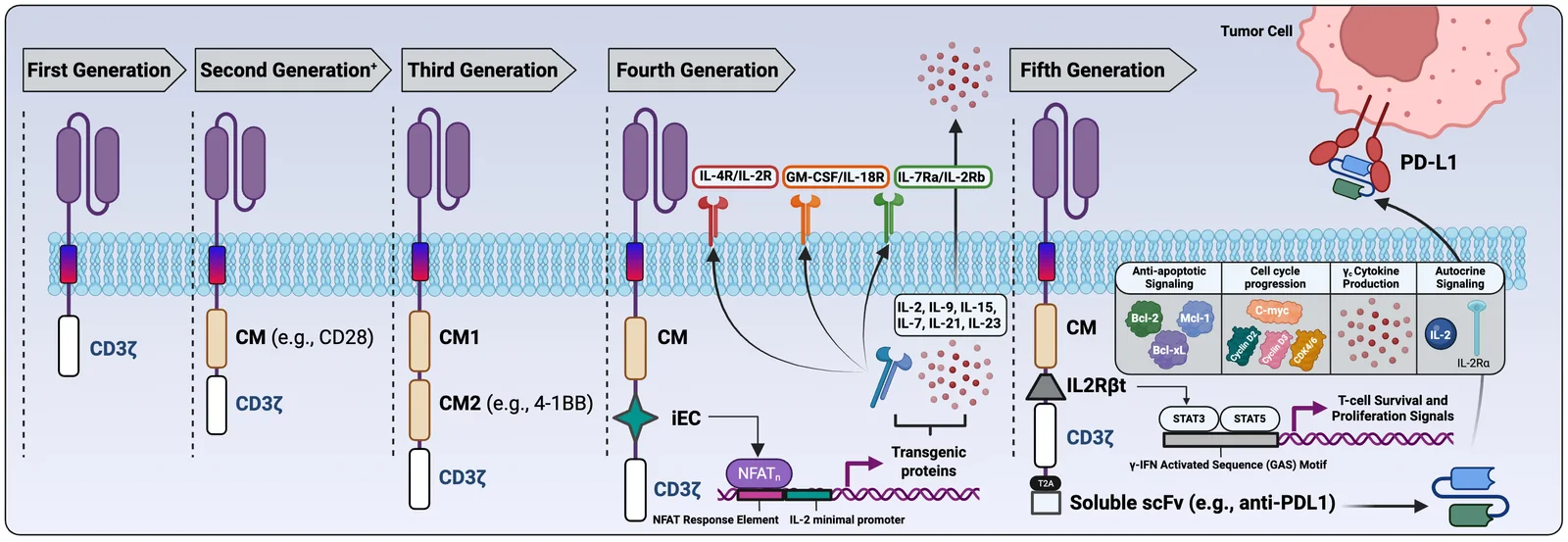
CARs through the generations. Schematic diagram of the evolution of CAR constructs through the generations. Briefly, first generation CARs are characterized by an extracellular ARD, a TMD, and a single signaling domain, CD3ζ. The second and third generation constructs incorporated one and two intracellular costimulatory molecules to this design, respectively. Fourth generation “TRUCKs” possess a single CM with an inducible expression cassette (iEC) which drives expression of transgenic proteins. Fifth “Next” generation CARs possess a single CM with a truncated intracellular IL-2Rβ chain for JAK/STAT signaling and T2A ribosomal skip sequences followed by scFv sequences for soluble scFv production. Created with biorender.com.

An early iteration of fifth generation CAR T-cells, described in 2018 by Kagoya and colleagues, was specifically engineered to promote optimal T-cell activation and proliferation. This was accomplished through the incorporation of cytokine receptor signaling domains into the endodomain of second-generation CAR T-cells, providing the critical “third signal” of T-cell activation via cytokine engagement. This construct involved the integration of a truncated cytoplasmic domain from the interleukin IL-2 receptor β-chain (IL-2Rβ) and a STAT3-binding tyrosine-X-X-glutamine (YXXQ) motif, which together enable antigen-dependent activation of the Janus Kinase (JAK) and Signal Transducer and Activator of Transcription (STAT) -3 and -5 transcription factor signaling pathways (i.e., release of γ_c_-cytokines in antigen-dependent manner) (95). More recently, in 2022, Yuti and colleagues generated their own fifth-generation CAR composed of murine anti-CD19 scFv, a CD8-HR, CD-28-TMD, three CMs (41BB-CD28-CD27) and CD3ζ signaling domain with an anti-PDL1 scFv cDNA sequence (derived from atezoluzumab) inserted downstream and linked to a T2A cleavage peptide, enabling the CAR to secrete anti-PDL1 in an antigen-dependent manner (96). Using this novel construct they were able to attenuate the PD-L1 meditated immune inhibition in aggressive B cell lymphoma cell lines (CD19^+/^PDL1^high^) and produce significant cytotoxicity at low effector:target (E:T) ratios (0.5:1). See **Figure 2** for more of an illustration of each CAR generation.

## 3 CNS malignancies and CAR T-cell therapy

### 3.1 Overview of CNS malignancies

CNS malignancies are notoriously difficult to treat lesions of the brain and spinal cord with a disproportionately high burden of morbidity and mortality relative to their incidence. Most cases arise secondary to metastatic spread of systemic cancers (i.e., secondary CNS malignancies via brain or leptomeningeal metastasis) with an estimated 40-50% arising from lung cancer, 15-30% from breast cancer, 5-20% from melanomas, 3-8% from colorectal cancer, and 2-4% from renal cell carcinomas (97). CNS involvement is often a harbinger of terminal systemic disease and is associated with dramatically worse prognoses across all primary cancer types.

Due to marked molecular and prognostic heterogeneity among systemic progenitor cancers, large retrospective studies and meta-analyses are helpful in identifying general trends associated with CNS involvement. Such studies reveal that following formation of CNS metastasis, median overall survival (in months) is reduced by as much as 61% in metastatic NSCLC (19.3 to 7.5) (98), 77% in metastatic HER2+/ER- breast cancer (34 to 7.9) (99, 100), 58% in metastatic melanoma (20.3 to 8.4) (101, 102), 89% in colorectal cancer (31.6 to 3.2) (103), and 57% in metastatic renal cell carcinoma (22.4 to 9.7) (101, 104). Despite the majority of CNS malignancies arising via metastases, an estimated 15% to 30% originate in the CNS (i.e., primary CNS malignancies) (105, 106). The fifth edition of the World Health Organization Classification of Tumors of the Central Nervous System (WHO CNS5), published in 2021, is the most up-to-date, comprehensive international reference for the classification and diagnosis of primary CNS malignancies. WHO CNS5 expanded upon past editions via the incorporation of advanced molecular diagnostics to the existing histological and immunohistochemical parameters, resulting in the introduction of two nosologically distinct categories of diffuse glioma, adult-type and pediatric-type to the pre-existing list including circumscribed astrocytic gliomas, ependymal tumors, embryonal tumor, and primary CNS lymphoma (107–110). Using these updated diagnostic criteria, GLOBOCAN estimates published in 2024 analyzing 36 different cancers across 185 countries found that primary CNS malignancies ranked 19^th^ in incidence with 321,476 new cases and 12^th^ in mortality with 248,305 deaths in 2022 (111–113). For comprehensive diagnostic criteria and epidemiological data concerning primary CNS malignancies, see **Table 2** (193, 194).

**Table 2:** Primary CNS Malignancies (WHO CNS5).

Tumor Family (All Ages)	Tumor Type	Diagnostic Criteria, Grade (1-4)^193^	Relative Survival (95% CI)^194^
Diffuse glioma	Glioblastoma, IDH-wildtype	IDH-WT and ≥ 1 of the following: microvascular proliferation, necrosis, TERT promoter mutation, EGFR amplification, or +7/-10 chromosome alterations, Grade 4	1-year RS: 43.0 (42.7-43.2) 5-year RS: 7.0 (6.8-7.1) 10-year RS: 4.4 (4.2-4.5)
	Astrocytoma, IDH-mutant, diffuse (D) and anaplastic (A)	IDH1 or IDH2 mutation, frequent ATRX and/or TP53 mutation AND absence of 1p/19q codeletion. (Grade 2–4); CDKN2A/B homozygous deletion automatically defines grade 4	1-year RS: D = 76.7 (76.3-77.4); A = 68.7 (67.9-69.4) 5-year RS: D = 53.7 (53.0-54.4); A = 33.6 (32.9-34.4) 10-year RS: D = 43.3 (42.5-44.1); A = 14.7 (13.9-15.5)
	Oligodendroglioma, IDH-mutant & 1p/19q-codeleted	IDH mutation and 1p/19q codeletion, Grade 2-3	1-year RS: 95.4 (94.9-95.7) 5-year RS: 85.2 (84.5-85.9) 10-year RS: 72.7 (71.6-73.8)
Circumscribed astrocytic gliomas	Pilocytic astrocytoma	Bipolar hair-like (pilocytic) cells, compact and loose or myxoid regions, Rosenthal fibres, and eosinophilic granular bodies; MAPK pathway alteration association (commonly *KIAA1549*::*BRAF* fusions), Grade 1	1-year RS: 98.1 (97.8-98.3) 5-year RS: 94.8 (94.4-95.2) 10-year RS: 93.1 (92.6-93.6)
Ependymal tumors	Ependymoma	Supratentorial: ZFTA–RELA fusion**;** Posterior fossa: PFA (H3K27me3 loss) or PFB (H3K27me3 retained), Grade 1-3	1-year RS: 95.9 (95.6-96.2) 5-year RS: 90.9 (90.4-91.4) 10-year RS: 87.2 (86.5-87.9)
Embryonal tumors	Medulloblastoma (WNT-activated, SHH-activated, Group 3/4)	Defined by molecular subgroup: CTNNB1 (WNT), SHH-pathway activation, MYC/MYCN amplification, Grade 4	1-year RS: 83.7 (83-84.4) 5-year RS: 65.4 (64.4-66.3) 10-year RS: 58.7 (57.7-59.8)
Meningeal tumors	Meningioma	Meningothelial tumor; possible TERT promoter mutation, or CDKN2A/B loss, Grade 1-3	1-year RS: 93.1 (93-93.1) 5-year RS: 88.2 (88.1-88.4) 10-year RS: 83.6 (83.4-83.9)

IDH, isocitrate dehydrogenase; WT, wildtype; RS, relative survival

### 3.2 Current landscape of CAR T-cell therapy in CNS malignancies

The formal adoption of CAR T-cells into the therapeutic anti-cancer arsenal has led to a modern renaissance in immuno-oncology. The clinical utility of genetically engineered immune effector cells has expanded far beyond hematologic malignancies, with potential indications such as solid tumors, autoimmune, and even neurodegenerative diseases. Early studies investigating CAR T-cell therapy for CNS malignancies provide an informative preview of the evolving clinical landscape. Through such a lens it is possible to identify major challenges associated with the modality. In this section we will discuss the challenges highlighted by preclinical and clinical investigations and present potential solutions currently in development.

#### 3.2.1 Trafficking and tumor infiltration

**Figure 3:**
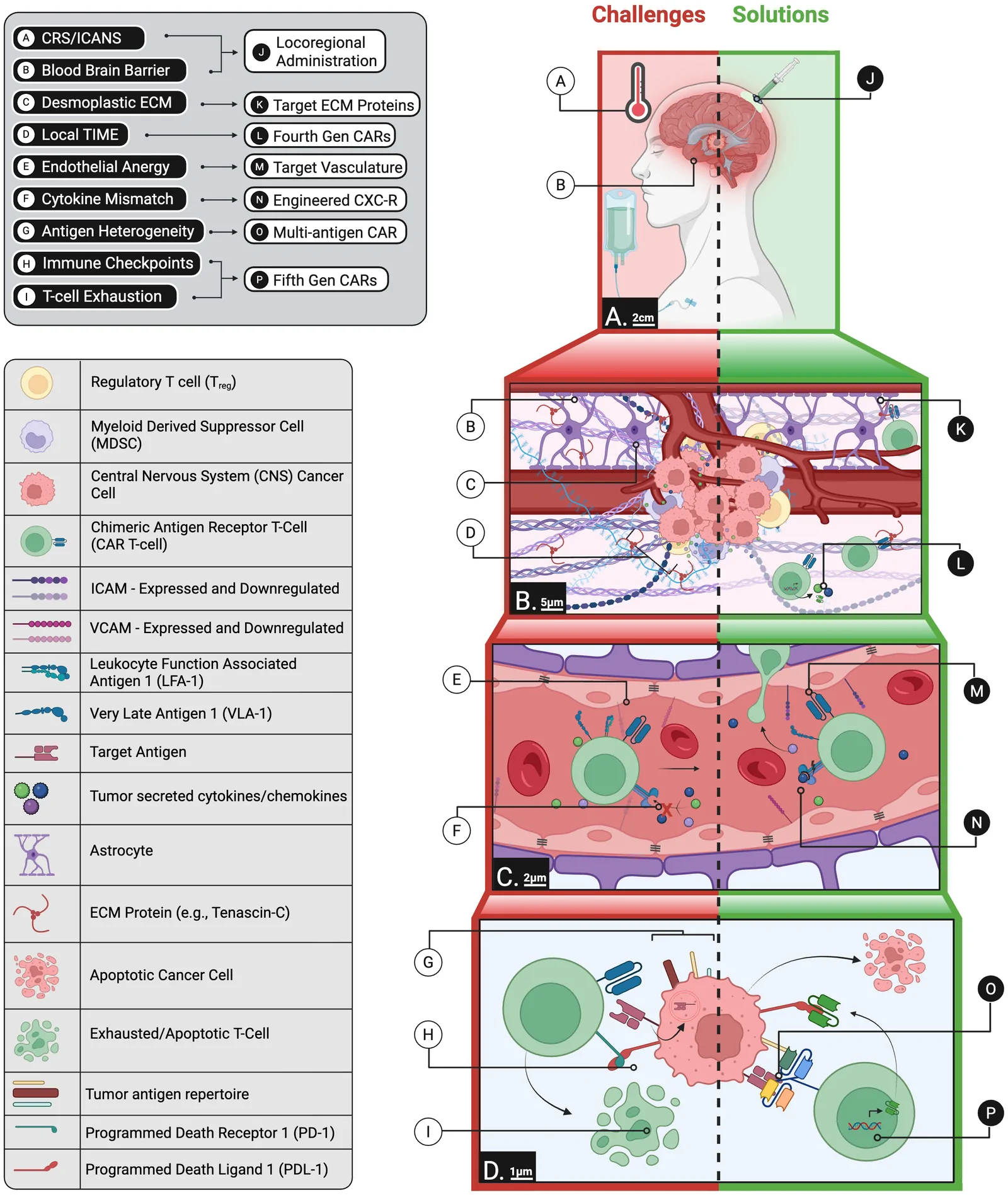
Current challenges and potential solutions associated with CAR T-cell therapy for CNS malignancies. Panels **(A–D)** represent a successively zoomed-in view of the dichotomy of major challenges associated with CAR-T cell therapy for CNS malignancies and their potential solutions. **(A)** Macroscopic challenges include the systemic toxicity syndromes that arise with CAR T-cell therapy and the structural barriers (BBB highlighted, though BCSFB and BTB are additional challenges) that must be overcome. Locoregional delivery of CAR T-cells is one option to simultaneously address both concerns. **(B)** At the level of the TIME, the BBB can be appreciated on a cellular level, alongside the desmoplastic ECM, and immunosuppressive milieu of cells and their cytokine mediators. The use of fourth generation CAR T-cells as well as specifically targeting ECM proteins (Tenascin-C highlighted) represent two approaches to circumvent these challenges. **(C)** Within the tumor vasculature the challenge presented by endothelial anergy and cytokine mismatch is made evident. Strategies to mitigate these concerns include targeting the tumor vasculature to increase expression of cell adhesion molecules, enabling CAR T-cell extravasation and designing CARs with modified chemokine receptors that correspond to those produced by the tumor. **(D)** At the level of CAR T-cell-Tumor engagement, intra-tumoral antigen heterogeneity is highlighted (though inter-tumor cell antigen heterogeneity is also a factor) as well as expression of immune checkpoint inhibitors that lead to CAR T-cell exhaustion. Employing fifth generation CAR T-cells that produce anti-PDL1 antibodies and designing CARs to target multiple antigens are potential strategies to avoid failure of CAR activation and exhaustion. Created with biorender.com.

For CAR T-cells to exercise their anti-tumor activity against CNS malignancies they must first bypass systemic and local barriers that prevent trafficking and infiltration of CARs into the tumor (**Figure 3**). Systemically, CAR-T cells must overcome structural barriers (i.e., the blood-brain barrier [BBB], blood-cerebrospinal fluid barrier [BCSFB], and blood-tumor barrier [BTB]) (114–116), chemokine mismatch (117, 118), and endothelial anergy (119). Locally, CARs must navigate the dynamic extracellular matrix of CNS malignancies, characterized by an extensive remodeling of glycoproteins, proteoglycans, and glycosaminoglycans that produce a dense, inflexible barrier (120, 121). Seeking to avoid these constraints, several research groups have begun to explore alternative routes of administration, such as intratumoral (ITm), intracavitary (ICT), intracerebroventricular (ICV), and intrathecal (IT). Numerous studies have demonstrated that locoregional delivery of CAR T-cells yields significant antitumor efficacy in glioblastoma, medulloblastoma, ependymoma and breast cancer brain metastases (122–129). Due to the targeted delivery to tumor sites, this locoregional approach has the potential to address multiple challenges simultaneously, avoiding all systemic barriers associated with intravenous delivery while minimizing on-target off-tumor toxicity and systemic side effects (130). Furthermore, reports from a recurrent high-grade glioma clinical trial (NCT02208362) have confirmed the safety of multiple-dose locoregional administration and its beneficial role in maintaining sufficient CAR T-cell levels at the malignant site (131, 132).

Chemokine mismatch and endothelial anergy are tumor-induced processes characterized by the secretion of chemokines that bind to receptors not normally expressed on T-cells and the reduction of adhesion molecule expression in tumor vasculature, respectively. As these concerns have contributed to the poor translational success of CAR T-cells from hematologic to solid tumors, developing strategies to obviate them are at the forefront of CAR T-cell research. In line with this mission, investigators have identified several chemokines and their cognate receptors (i.e., CXCL9, CXCL10, and CXCL11 binding CXCR3 (133); CXCL13 binding CXCR5 (134); CCL17 and CCL22 binding CCR4 (135, 136); CCL2, CCL7, CCL8, and CCL13 binding CCR2 (137); and CX3CL1 binding CX3CR1 (138, 139)) as well as adhesion molecules (i.e., CD49a and CD49d integrins, P- and E-selectins, ICAM-1 and VCAM-1) (140, 141) essential for CAR T-cell trafficking into the CNS. Engineering CAR T-cells to express these chemokine receptors can dramatically reduce the impact of chemokine mismatch (59, 142). For example, Jin and colleagues demonstrated that constructing CAR T-cells to express CXCL8 receptors (CXCR1 or CXCR2) resulted in significantly enhanced intratumoral trafficking and persistence of T-cells in preclinical models of glioblastoma, ovarian and pancreatic cancer, ultimately resulting in complete tumor regression and long-lasting immunologic memory (143). Additionally, Song and colleagues demonstrated superior tumor trafficking and anti-tumor efficacy of intracerebroventricularly administered B7-H3 CAR T-cells engineered to overexpress CXCR3-A (a CXCL10 receptor) in a diffuse intrinsic pontine glioma (DIPG) mouse model (144).

While not specific to CNS malignancies, data from systemic cancers suggest targeting tumor vasculature may be an effective strategy to overcome the effects of endothelial anergy (145, 146). For example, Winkler and colleagues demonstrated a “vascular normalization” in brain tumors following VEGFR2 blockade which enhanced the efficacy of subsequent radiation and chemotherapy due to improved oxygenation and local drug delivery, respectively (147). Building upon these data, Chinnasamy and colleagues demonstrated that pretreatment with CAR T-cells targeting VEGFR-2 enhanced trafficking and anti-tumor efficacy of subsequently administered CAR T-cells targeting tumor specific antigens (PMEL, TRP1, and TRP2) in a B16 melanoma mouse model (148).

The final physical barrier impeding access to solid tumors is the stroma (i.e., the supportive, non-cancerous tissue encasing the tumor). The stroma is characterized by a desmoplastic ECM that limit the infiltrative capacity of CAR T-cells (149). Modifying CAR T-cells to target ECM structural proteins, such as Tenascin-C (150), a glycoprotein involved in cellular adhesion overexpressed in glioblastoma, or fibroblast activating protein (FAP) (151), a biomarker for cancer related fibroblasts, have shown promise in improving tumor infiltration. Additionally, engineering CAR T-cells to overexpress ECM-degrading enzymes such as heparanase has likewise been found to facilitate improved tumor infiltration (152). Finally, studies investigating the synergistic effects of radiation therapy (RT) and CAR T-cell administration in glioblastoma models demonstrate that radiation-induced ECM degradation and increased BBB permeability can improve overall antitumor efficacy (153).

#### 3.2.2 Local tumor immune microenvironment

An immune cell’s ability to recognize a molecular signature as “self” or “non-self” and selectively activate in response to “non-self” recognition (i.e., self-tolerance) is a hallmark of the mammalian immune system. Self-tolerance develops at various stages of immune cell maturation, with early central-tolerance selection processes occurring in the bone marrow and thymus (for B-cell and T-cell lineages, respectively) and later exposure to peripheral-tolerance mechanisms, such as inhibitory immune checkpoints (i.e., PD-L1/2, CD80/86 expression), activation induced cell death (AICD), costimulatory signal-dependent activation, and immunosuppressive cell populations such as T-regulatory cells (Treg), tumor-associated macrophages (TAMs) and myeloid-derived suppressor cells (MDSC) (154). While critical to ensuring the host immune system does not attack and destroy itself, these tolerance mechanisms are co-opted and leveraged by solid tumors to evade the immune system (**Figure 3**). Given the profound functional consequences of an aberrant immune response within the CNS, immune tolerance mechanisms are amplified in the CNS. To ensure a quiescent immune environment is maintained, redundant tolerance mechanisms such as astrocytic immune regulation via PDL1 expression and TGF-β secretion (155), myeloid cell reprogramming to immune suppressing phenotypes, and Treg enrichment have evolved (156). The complexity of neuroimmune interactions with cancer cells demands a multidisciplinary approach to determine the best therapeutic modality. Fourth and fifth generation CAR constructs will likely become useful in this context as they are specifically designed to combat the immunosuppressive tumor immune microenvironment (TIME) of solid tumors. Indeed, preclinically tested strategies to overcome the TIME in the CNS include generating CARs with PD-1 KO and IL-15 expression (85, 157, 158). Additionally, the *ex vivo* culture conditions of CAR T-cells has been shown to influence *in vivo* effector states, allowing researchers to optimize cellular differentiation through fine-tuned manufacturing protocols (39, 159).

#### 3.2.3 Antigen heterogeneity

In addition to their uniquely immunosuppressive TIME, CNS malignancies also exhibit substantial antigen heterogeneity (160). As conventional CAR T-cell therapies rely on targeting a single antigen, this property of CNS malignancies dramatically increase the risk of antigen escape, a phenomenon in which the targeted antigen is downregulated by the tumor making it invisible to the CAR T-cells. Antigen escape can occur through several mechanisms, including alterations in the antigen’s gene expression (due to mutations or alternative splicing) (161, 162), deficiencies in antigen processing or presentation (163), lineage switch (164, 165), antigen loss through trogocytosis (166), and clonal proliferation of pre-existing target-negative tumor cells post-treatment (167). Several strategies incorporating multiple antigen targets (i.e., tandem, dual/multi, synNotch, and logic-gated constructs) are under investigation and have produced promising results in preclinical and clinical studies (168, 169). Antigens that have been extensively studied and validated as effective targets for CAR T-cell therapy include human epidermal growth factor receptor 2 (HER2/ErbB2), ephrin type-A receptor 2 (EphA2), epidermal growth factor receptor variant III (EGFRvIII), EGFR806, chlorotoxin, CD70, NKG2D, CD276 (B7-H3), disaloganglioside (GD2), and Interleukin-13 receptor subunit alpha-2 (IL-13Rα2) (122, 125, 128, 131, 170) (171, 172). See **Table 3** for more details on these antigen targets.

**Table 3:** Validated antigen targets for CNS malignancies.

Antigen	Molecular features	scFv clones	Rationale	Cancer types	Clinical trials
HER2	MW: ~185 kDa Class: RTK	FRP5 (most common); 4D5, trastuzumab derived	Lowly expressed in normal brain tissue, overexpressed in CNS cancers	GBM, MB, EPN, BCBM	NCT03389230, NCT04903080, NCT02442297, NCT05768880, NCT03500991, NCT03696030, NCT01109095
EphA2	MW: ~130 kDa Class: RTK	4H5	Lowly expressed in normal brain tissue, overexpressed in CNS cancers, promotes tumor growth, angiogenesis, invasion	GBM	NCT034239
EGFRvIII	MW: ~145 kDa Class: mutant RTK, del(2-7)EGFR	3C10; 139; MR1	Tumor-specific neoantigen, not expressed in healthy tissue, constitutively active, promotes tumor growth	GBM	NCT07244666, NCT05802693, NCT05660369, NCT05063682, NCT03726515, NCT03283631, NCT02844062, NCT02664363, NCT02209376, NCT03423992, NCT01454596
EGFR806	MW: ~170 kDa Class: RTK	806, derived from mAb806	Tumor-specific cryptic epitope (located in extracellular domain II of EGFR), amplified in GBM	GBM	NCT05768880, NCT03638167
B7H3	MW: ~100 kDa Class: Ig B7 superfamily ligand, type I transmembrane protein	376.96 (most common); MGA271 variants derived from mAb	Lowly expressed in normal brain tissue, overexpressed in GBM, expressed on cancer stem cells	DMG, GBM, MB	NCT07193628, NCT06018363 NCT05474378, NCT05366179 NCT05241392, NCT04385173 NCT04077866, NCT06737146 NCT06482905, NCT05752877 NCT06221553, NCT05835687 NCT05768880, NCT04185038 NCT06742593, NCT06592092
GD2	MW: Variable Class: Disialoganglioside	14.G2a (most common); ch14.18-derived	Highly expressed on aggressive malignancies (especially H3-K27M mutant types). Expression associated with tumor proliferation, invasion, stemness	DMG, GBM, NBM	NCT06764537, NCT04406610, NCT03423992, NCT05298995, NCT04196413, NCT04099797
IL13Rα2	MW: ~65 kDa Class: Type I transmembrane cytokine receptor	IL13 mutein (E13Y) ligand-based binder (not scFv, most common); mAb47 variants	Highly tumor-restricted in GBM, minimally expressed in healthy tissue	DMG, GBM, LGG	NCT07209241, NCT07193628, NCT06973096, NCT05168423, NCT04003649, NCT02208362, NCT05752877, NCT05540873, NCT03423992, NCT06815029, NCT04661384, NCT04510051
CD70	MW: ~30 kDa Class: TNF-family ligand	41D12 (lymphoma derived scFv)	Highly tumor-restricted in GBM, minimally expressed in healthy tissue	GBM	NCT05353530, NCT06946680, NCT06828341
Chlorotoxin (MMP2 complex)	MW: Variable Class: ECM-associated receptor complex	Chlorotoxin peptide binder (not scFv)	High binding to GBM, low binding to healthy brain	GBM	NCT05627323, NCT04214392
NKG2D	MW: ~40–60 kDa Class: stress-induced ligand, “danger signal”, includes MIC and ULBP families	NKG2D receptor ectodomain (not scFv)	Highly expressed on tumor and immunosuppressive cell surface, low expression in healthy brain	GBM	NCT04717999
CD19	MW: ~95 kDa Class: B-cell surface protein	FMC63	Highly expressed on CNS lymphoma	CNSL	NCT07137494, NCT07062627, NCT05651178, NCT05625594, NCT04608487

MW, molecular weight; RTK, receptor tyrosine kinase; BCBM, breast cancer brain metastases; DMG, diffuse midline glioma; LGG, low-grade glioma; MB, medulloblastoma; EPN, ependymoma; NBM, neuroblastoma metastasis; CNSL, CNS Lymphoma.

#### 3.2.4 CAR T-cell related toxicities within the CNS

Despite significant resources having already been invested into optimizing the safety of CAR T-cell therapy for hematologic malignancies, the serious, potentially fatal consequences of even mild toxicity to the delicate structures of the CNS necessitate further fine-tuning of CAR safety. Preclinical and clinical studies demonstrate that both “on target/off tumor” (i.e., CRS and ICANS) and “on target/on tumor” (tumor inflammation-associated neurotoxicity, TIAN) toxicity syndromes can occur when targeting CNS malignancies with CAR T-cells. Together, these adverse drug reactions (ADRs) narrow the therapeutic index of the modality, requiring inpatient monitoring and limiting the patient populations that can benefit from it. In this section we will discuss the basic pathophysiology of the toxicity syndromes and highlight promising avenues being explored to minimize their impact. For details regarding toxicities reported in clinical trials, see **Table 4**.

**Table 4:** CAR T-cell clinical trials in CNS malignancies.

NCT	Study title	CAR ID/Construct details	Delivery	Dose	Location	Enrolled^+^	Status and results
Glioblastoma Multiforme (Adult and Pediatric)							
NCT07244666	Safety and Preliminary Efficacy of a Metabolically Armed Chimeric Antigen Receptor T Cell Therapy Targeting EGFRvIII for Recurrent Glioblastoma	ID: Meta10-EGFRvIII or LMC005 scFv: anti-EGFRvIII HR/TMD: NR CM: NR Armoring: IL-10 expression cassette	ICV and IV	NR	Zhejiang University, China	36	Not yet recruiting; no results
NCT07209241	Different Approaches for CART-EGFR-IL13Ra2 Dosing in Recurrent GBM	ID: CART-EGFR-IL13Ra2 scFv: anti*-m*EGFR-*hIL13Ra2* HR/TMD: NR CM: 4-1BB	Varies	NR	University of Pennsylvania, USA	12	Recruiting; no results
NCT07193628	B7H3/IL13Ra2 Bispecific Armored Chimeric Antigen Receptor T-Cell Therapy Study for Recurrent/Refractory Glioblastoma	ID: EPC-003 scFv: anti-B7H3/IL13Ra2 HR/TMD: NR CM: NR	ICV (Omaya reservoir)	DL1: 2.5 x 10^6^ DL2: 5.0 x 10^6^ DL3: 10 x 10^6^ DL4: 20 x 10^6^	Zhejiang University, China	14	Recruiting; No results
NCT07180927	DLL3 CAR-T Therapy Targeting Brain Tumors	ID: 4sCAR-DLL3 scFv: anti-DLL3 HR/TMD: NR CM: NR	IV	1x 10^6^ cells/kg BW	Shenzhen Geno-Immune Medical Institute, China	30	Recruiting; No results
NCT06973096	CART-EGFR-IL13Ra2 in Newly Diagnosed GBM Following Initial Radiotherapy	ID: CART-EGFR-IL13Ra2 scFv: anti*-m*EGFRe806-*hIL13Ra2* HR/TMD: NR CM: 4-1BB	ICV	DL1: 2.5 x 10^7^ DL-1: 1.0 x 10^7^	University of Pennsylvania, USA	9	Recruiting; No results
NCT06815432	GPC-3 CAR T CELLS FOR Recurrent GPC-3 Positive Glioblastoma	ID: 15.GPC3-CAR T cells scFv: anti-GPC3 HR/TMD: NR CM: NR Armoring: IL-15 expression cassette Safety: iCasp9 via AP1903	ICV (Omaya reservoir)	DL1: 5.0 x 10^6^ DL2: 10 x 10^6^ DL3: 50 x 10^6^ DL4: 100 x 10^6^	Baylor College of Medicine, USA	27	Recruiting; No results
NCT06764537	Evaluation of in Vitro Antitumor Activity of GD2 CAR-T Cells in Glioblastoma “BOOSTCAR”	ID: GD2 CAR-T Cells scFv: anti-GD2 HR/TMD: NR CM: NR	NA	NA	Central Hospital, Nancy, France	18	Not yet recruiting; no results
NCT06616727	The Safety and Efficacy of SNC-109 CAR-T Cells Therapy the rGBM	ID: SNC-109 CAR-T Cell (Dual) scFv: NR HR/TMD: NR CM: NR	NR	DL1: 5.0 x 10^4^ + escalation	Shanghai Simnova Biotechnology Co., Ltd., China	50	Enrolling by invitation; No Results
NCT06018363	Clinical Study on the Treatment of Malignant Brain Glioma by QH104 Cell Injection	ID: Allogenic B7-H3 CAR-γδT cell (QH104) scFv: anti-B7-H3 HR/TMD: NR CM: NR	ICV (Omaya reservoir) or IT	DL1: 1.0 x 10^7^ DL2: 3.0 x 10^7^ DL3: 6.0 x 10^7^	Dushu Lake Hospital Affiliated to Soochow University, China	25	Recruiting; No results
NCT05868083	The Safety and Efficacy of SNC-109 CAR-T Cells Therapy the Recurrent Glioblastoma	ID: SNC-109 CAR-T Cell (Dual) scFv: NR HR/TMD: NR CM: NR	NR	DL1: 2.0 x 10^4^ + escalation	Shanghai Simnova Biotechnology Co., Ltd., China	16	Unknown Status; No Results
NCT05802693	A Study to Evaluate the Safety, Tolerance and Initial Efficacy of EGFRvIII CAR-T on Glioblastoma	ID: DCTY0801 Autologous T lymphocyte scFv: anti-EGFRvIII (c806) HR/TMD: NR CM: 4-1BB	ICV (Omaya reservoir)	DL1: 22 x 10^6^ DL2: 60 x 10^6^ DL3: 160 x 10^6^ DL4: 220 x 10^6^	Beijing Tsinghua Chang Gung Hospital, China	22	Unknown Status; No Results
NCT05660369	CARv3-TEAM-E T Cells in Glioblastoma	ID: CARv3-TEAM-E T cells scFv: anti-EGFRvIII HR/TMD: NR CM: 4-1BB Armoring: EGFR-CD3 TEAM	ICV (Omaya reservoir)	Safety Run DL1: 10 x 10^6^	Massachusetts General Hospital, USA	21	Interim Results;^129^ DL1; n=3 CRS – 3/3 (100%); Grade 1-2 ICANS – 3/3 (100%); Grade 2 TIAN – 3/3 (100%); Grade 1-2
NCT05627323	CAR T Cells in Patients With MMP2+ Recurrent or Progressive Glioblastoma	ID: CHM-1101 CAR-T cells scFv: Chlorotoxin (from deathstalker scorpion) HR/TMD: CD8a/CD8a CM: CD28	ICT (Rickham catheter) or ICV	NR	City of Hope Hospital, USA and St. David’s South Austin Medical Center, USA (Chimeric Therapeutics)	42	Active, Not Recruiting; No Results
NCT05577091	Tris-CAR-T Cell Therapy for Recurrent Glioblastoma	ID: Autologous Tris-CAR-T cell (Dual) scFv: anti-CD44/CD133 HR/TMD: NR CM: NR Armoring: IL-7Ra	ITm or ICV (Omaya reservoir)	DL0: 1.0 x 10^7^ DL1: 1.0 x 10^8^ DL2: 5.0 x 10^7^	Beijing Tiantan Hospital, China	10	Recruiting; Interim Results (195); n=4 No neurotoxicity or AE greater than Grade 2 observed; radiographic tumor regression or suppression observed within days
NCT05474378	B7-H3 Chimeric Antigen Receptor T Cells (B7-H3CART) in Recurrent Glioblastoma Multiforme	ID: B7-H3CART scFv: anti-B7H3 HR/TMD: NR CM: NR	ICV or ICV+ITm	DL-1: 5 x 10^6^ DL1:10 x 10^6^ DL2: 25 x 10^6^ DL3: 50 x 10^6^ DL4: 100 x 10^6^	Stanford University, USA	39	Recruiting; Interim Results (196); n=11 TIAN observed in 29/36 (81%) infusions Median OS = 14.6 months
NCT05366179	Autologous CAR-T Cells Targeting B7-H3 in Recurrent or Refractory GBM CAR.B7-H3Tc	ID: CAR.B7-H3 T cells scFv: anti-B7H3 HR/TMD: NR CM: NR	ICV	DL1: 2 x 10^6^ DL2: 5 x 10^6^	UNC Lineberger Comprehensive Cancer Center, USA	36	Recruiting; No Results
NCT05353530	IL-8 Receptor-modified CD70 CAR T Cell Therapy in CD70+ Adult Glioblastoma “IMPACT”	ID: 8R-70CAR T-cell scFv: anti-CD70 HR/TMD: NR CM: NR Armoring: IL8R	IV	DL1: 1 x 10^6^ DL2: 10 x 10^6^ DL3: 100 x 10^6^	University of Florida, USA	39	Recruiting; No Results
NCT05241392	Safety and Efficacy Study of Anti-B7-H3 CAR-T Cell Therapy for Recurrent Glioblastoma	ID: B7-H3-targeting CAR-T cells scFv: anti-B7H3 HR/TMD: NR CM: NR	ITm or ICV (Omaya reservoir)	DL1: 20 x 10^6^ DL2: 60 x 10^6^ DL3: 150 x 10^6^ DL4: 450 x 10^6^ DL5: 900 x 10^6^	Beijing Tiantan Hospital, China	30	Active, Not Recruiting; Interim Results (197); n=13 13/13 (100%) experienced at least 1 AE: CRS, increased ICP, HA, epilepsy, decreased LOC, vomiting, and pyrexia; Grade 1-3 Median OS = 20.3 months
NCT05168423	CART-EGFR-IL13Ra2 in EGFR Amplified Recurrent GBM	ID: CART-EGFR-IL13Ra2 scFv: anti*-m*EGFRe806-*hIL13Ra2* HR/TMD: NR CM: 4-1BB	IT	DL-1: 5 x 10^6^ DL1: 10 x 10^6^ DL2: 25 x 10^6^ DL3: 50 x 10^6^	University of Pennsylvania, USA	67	Interim Results (198); DL1; n=3 and DL2; n=3 CRS – 6/6 (100%); Grade 1-2 ICANS – 6/6 (100%); Grade 2-3 TIAN – 6/6 (100%); Grade 2-3 Tumor shrinkage in all 6, up to 30% in 3/6
NCT05063682	A Phase 1 Study to Evaluate EGFRvIII -Targeted Chimeric Antigen Receptor (CAR) T Cells for Adult Patients With Leptomeningeal Glioblastoma “CARTREMENDOUS”	ID: EGFRvIII -CAR T cells scFv: anti-EGFRvIII HR/TMD: NR CM: 4-1BB	ICV	NR	Jyväskylä Central Hospital, India	10	Unknown Status; No Results
NCT04717999	Pilot Study of NKG2D CAR-T in Treating Patients With Recurrent Glioblastoma	ID: UWN2D CAR-T scFv: anti-NKG2D HR/TMD: NR CM: NR	ICV (Omaya reservoir)	NR	UWell Biopharma	20	Unknown Status; No Results
NCT04385173	Pilot Study of B7-H3 CAR-T in Treating Patients With Recurrent and Refractory Glioblastoma	ID: BP102 scFv: anti-B7-H3 HR/TMD: NR CM: CD28	ITm or ICV (Omaya reservoir)	NR	Second Affiliated Hospital, School of Medicine, Zhejiang University, China	12	Unknown Status; No Results
NCT04214392	Chimeric Antigen Receptor (CAR) T Cells With a Chlorotoxin Tumor-Targeting Domain for the Treatment of MMP2+ Recurrent or Progressive Glioblastoma	ID: Chlorotoxin-CD28-CD3z-CD19t-expressing CAR T-cells scFv: Chlorotoxin (from deathstalker scorpion) HR/TMD: CD8a/CD8a CM: CD28	ICT, ICV, or ICT and ICV dual delivery	NR	City of Hope Medical Center, USA	19	Active, Not Recruiting; Interim Results (199); n=4 No CNS-related AE > Grade 3; 2/4 (50%) with Grade 3 Cerebral Edema Overall DCR = 75%
NCT04077866	B7-H3 CAR-T for Recurrent or Refractory Glioblastoma	ID: B7-H3 CAR-T scFv: anti-B7-H3 HR/TMD: NR CM: NR	ITm or ICV (Omaya reservoir)	NR	Second Affiliated Hospital, School of Medicine, Zhejiang University, China	40	Unknown Status; No Results
NCT04045847	CD147-CART Cells in Patients With Recurrent Malignant Glioma.	ID: CD147-CART scFv: anti-CD147 HR/TMD: NR CM: NR	ICT	NR	Xijing Hospital, China	31	Unknown Status; No Results
NCT04003649	IL13Ra2-CAR T Cells With or Without Nivolumab and Ipilimumab in Treating Patients With GBM	ID: IL13 [EQ]BBzeta/truncated CD19[t]+ TN/MEM Cells scFv: anti-IL13Ra2 HR/TMD: NR CM: 4-1BB	ICV or ITm	NR	City of Hope Medical Center, USA	60	Recruiting; No Results
NCT03726515	CART-EGFRvIII + Pembrolizumab in GBM	ID: CART-EGFRvIII T cells scFv: anti-hEGFRvIII HR/TMD: CD8/CD8 CM: 4-1BB	NR	NR	University of Pennsylvania, USA	7	Completed (200); n=7 No DLT; 1/7 (14%) with cerebral edema Grade 3-4; 1/7 (14%) with cognitive disturbance Grade 1-2; 1/7 (14%) with Dysphasia Grade 1-2; 1/7 (14%) with encephalopathy Grade 3-4; 1/7 (14%) with facial muscle weakness Grade 1-2; 1/7 (14%) with weakness Grade 3-4; 1/7 (14%) with seizure Grade 3-4; 6/7 (86%) with HA Grade 1-2 Median PFS = 5.2 months Median OS = 11.8 months
NCT03283631	Intracerebral EGFR-vIII CAR-T Cells for Recurrent GBM “INTERCEPT”	ID: Gamma-retroviral MSGV1–139 scFv EGFRvIII CAR gene-modified T cells scFv: anti-EGFRvIII HR/TMD: NR CM: NR	ICV	250 x 10^6^	Duke University, USA	2	Terminated; No Results
NCT03170141	Immunogene-modified T (IgT) Cells Against Glioblastoma Multiforme	ID: Antigen-specific IgT cells (4^th^ gen) scFv: anti-GD2 HR/TMD: CD28 CM: CD28 and 4-1BB Safety: iCasp9	IV	5 x 10^5^/kg BW to 5 x 10^6^/kg BW	Shenzhen Geno-Immune Medical Institute, China	30	Enrolling by invitation; Interim Results (201); n=8 1/8 (12.5%) with grade 3 seizure and grade 2 HA; no other CAR-related AE Median OS = 10 months
NCT02937844	Pilot Study of Autologous Chimeric Switch Receptor Modified T Cells in Recurrent Glioblastoma Multiforme	ID: Anti-PD-L1 CSR T cells scFv: anti-PDL1 HR/TMD: CD28 CM: CD28	IV	5 x 10^4^/kg to 1 x 10^7^/kg	Beijing Sanbo Brain Hospital, China	20	Unknown Status; No Results
NCT02844062	Pilot Study of Autologous Anti-EGFRvIII CAR T Cells in Recurrent Glioblastoma Multiforme	ID: anti-EGFRvIII CAR T cells scFv: anti-EGFRvIII HR/TMD: NR CM: NR	IV	5 x 10^4^/kg to 1 x 10^7^/kg	Beijing Sanbo Brain Hospital, China	20	Unknown Status; No Results
NCT02664363	EGFRvIII CAR T Cells for Newly Diagnosed WHO Grade IV Malignant Glioma “ExCeL”	ID: EGFRvIII CARs scFv: anti-EGFRvIII HR/TMD: NR CM: CD28 and 4-1BB (3^rd^ gen)	IV	DL1: 4.5 x 10^6^/kg DL2: 1.5 x 10^7^/kg DL3: 4.5 x 10^7^/kg DL4: 1.5 x 10^8^/kg	Duke University, USA	3	Completed; n=3 Preclinical Data Reported (202) 0/3 (0%) with DLT 1/3 (33.3%) with significant AE; 1/3 (33.3%) with cognitive disturbance, dizziness, or dysphagia
NCT02209376	Autologous T Cells Redirected to EGFRVIII-With a Chimeric Antigen Receptor in Patients With EGFRVIII+ Glioblastoma	ID: CART-EGFRvIII scFv: anti-EGFRvIII HR/TMD: CD8/CD8 CM: 4-1BB	IV	1 x 10^8^ to 5 x 10^8^	University of Pennsylvania, USA	11	Completed (172); n=10 received CARs No off tumor toxicity or CRS reported; A total of 54 CAR-related AE were reported; 24 were Grade 1; 21 were grade 2; 7 were grade 3; 2 were grade 4 (cerebral edema) Median OS = 251 days (8.2 months)
NCT02208362	Genetically Modified T-cells in Treating Patients With Recurrent or Refractory Malignant Glioma	ID: IL13Ra2-CAR/CD19t+ Tcm scFv: anti-IL13Ra2 HR/TMD: NR CM: 4-1BB	ICT, ICV, or ICT and ICV dual delivery	22 x 10^6^ to 220 x 10^6^	City of Hope Medical Center, USA	65	Completed (122, 131); n=65, n=58 evaluated for OS, AE, response Toxicity ≥ Grade 3 observed in 40% (n=23) with n=1 encephalopathy (Grade 3); n=1 ataxia (Grade 3); n=2 cerebral edema (Grade 4) n=29 (50%) achieved SD or better and n=13 (22%) achieved cSD or better Median OS = 8 months
NCT01109095	CMV-specific Cytotoxic T Lymphocytes Expressing CAR Targeting HER2 in Patients With GBM “HERT-GBM”	ID: HER.CAR CMV-specific CTLs scFv: anti-HER2 HR/TMD: NR CM: CD28	IV	DL1: 1 x 10^6^/m^2^ DL2: 3 x 10^6^/m^2^ DL3: 10 x 10^6^/m^2^ DL4: 30 x 10^6^/m^2^ DL5: 100 x 10^6^/m^2^	Baylor College of Medicine, USA	16	Completed (190); n=16 8/16 (50%) with PD; 8/16 (50%) with objective response 1/16 (6.25%) with PR of 62% TVR for 8 months; 7/16 (44%) with SD for > 6 weeks; 3/16 (19%) followed up for > 30 months Median OS post-infusion = 11.6 months; Median OS post-dx = 24.8 months
Mixed (GBM + other HGG) Primary CNS Malignancies							
NCT06964737	Anti-GARP Chimeric Antigen Receptor T Cell Therapy for the Treatment of Recurrent Grade III or IV Gliomas	ID: Anti-GARP CAR T Cells scFv: anti-GARP HR/TMD: NR CM: NR	ICT	NR	The Ohio State University Comprehensive Cancer Center, USA	30	Recruiting; No Results Preclinical Publication (203)
NCT06946680	IL-8 Receptor-modified CD70 CAR T Cell Therapy in CD70+ Pediatric High-grade Glioma (HGG)	ID: 8R-70CAR T cells scFv: anti-CD70 HR/TMD: NR CM: NR Armoring: IL8R (CXCR2) gene cassette	IV	DL1: 1 x 10^6^/kg DL2: 10 x 10^6^/kg DL3: 100 x 10^6^/kg	University of Florida, USA	18	Recruiting; No Results
NCT06828341	A Single-arm, Open, Exploratory Clinical Study of Allogeneic CAR-T Cells in the Treatment of Relapsed/Refractory Brain Gliomas With Positive CD70 Expression	ID: allogeneic CAR-T targeting CD70 scFv: anti-CD70 HR/TMD: NR CM: NR	IT	1 x 10^6^/kg	Jinling Hospital, China	3	Recruiting; No Results
NCT06737146	Study of MT027 in Patients with Recurrent or Progressive High-grade Glioma	ID: MT027 scFv: anti-B7H3 HR/TMD: NR CM: 4-1BB	NR	DL1: 1 x 10^7^ DL2: 3 x 10^7^ DL3: 6 x 10^7^	Tongji Hospital affiliated to Tongji Medical College of Huazhong University of Science & Technology, China	12	Not yet Recruiting; No Results
NCT06482905	Safety and Efficacy Study of TX103 CAR-T Cell Therapy for Recurrent or Progressive Grade 4 Glioma.	ID: TX103 scFv: anti-B7H3 HR/TMD: NR CM: NR	ICV or ICT	DL1: 6 x 10^7^ DL2: 1.5 x 10^8^ DL3: 2.5 x 10^8^	Mayo clinic in Arizona, Florida, Rochester, USA Beijing Tiantan Hospital, China	52	Recruiting; No Results
NCT05752877	Safety and Efficacy of Targeted IL-13Ra2 or B7-H3 UCAR-T for Advanced Glioma	ID: 13Rα2 or B7-H3 UCAR-T cell scFv: anti-IL13Rα2 or anti-B7-H3 HR/TMD: NR CM: NR	ICV or ICT	1 x 10^7^ to 5 x 10^7^	Second Affiliated Hospital of Soochow University, China	12	Recruiting; No Results
NCT05540873	A Clinical Study of IL13Ra2 Targeted CAR-T in Patients With Malignant Glioma	ID: YYB-103 scFv: anti-IL13Rα2 HR/TMD: NR CM: NR	IV	NR	National Cancer Center, Korea	18	Unknown Status; Interim Results (204); n=10 No DLT observed; all AE ≤ grade 3 n=9 available for survival analysis; Median PFS = 2.6 months; median OS = 10.9 months
NCT04406610	CAR-T Cell Immunotherapy for GD2 Positive Glioma Patients	ID: GD2 CAR-T scFv: anti-GD2 HR/TMD: NR CM: NR	IV	NR	Fuda Cancer Hospital, Guangzhou, China	0	Withdrawn; no results
NCT03423992	Personalized Chimeric Antigen Receptor T Cell Immunotherapy for Patients With Recurrent Malignant Gliomas	ID: NA scFv: anti-EGFRvIII, anti-IL13Rα2, anti-Her-2, anti-CD133, anti-EphA2, anti-GD2 HR/TMD: CD8/CD8 CM: 4-1BB Safety: tEGFR	NR	NR	Xuanwu Hospital, Beijing, China	100	Unknown Status; Interim Results (205); n=3 CRS – 2/3 (66.6%); Grade 2 OS: 86 to 181 days
NCT03389230	Memory-Enriched T Cells in Treating Patients With Recurrent or Refractory Grade III-IV Glioma	ID: HER2(EQ)BBζ/CD19t+ T cells scFv: anti-HER2 HR/TMD: NR CM: 4-1BB	ITm, ICT, or dual	NR	City of Hope Medical Center, USA	29	Active, not recruiting; No Results
NCT02617134	CAR-T Cell Immunotherapy in MUC1 Positive Solid Tumor	ID: anti-MUC1 CAR-T cells scFv: anti-MUC1 HR/TMD: NR CM: NR	NR	NR	PersonGen BioTherapeutics (Suzhou) Co., Ltd., China	20	Unknown status; No Results
NCT02331693	CAR T Cells in Treating Patients With Malignant Gliomas Overexpressing EGFR	ID: anti-EGFR CAR T scFv: anti-EGFR HR/TMD: NR CM: NR	NR	NR	Shanghai Cancer Institute, China	10	Unknown status; No Results
NCT01454596	CAR T Cell Receptor Immunotherapy Targeting EGFRvIII for Patients With Malignant Gliomas Expressing EGFRvIII	ID: EGFRvIII CAR scFv: anti-EGFRvIII HR/TMD: CD8/CD8 CM: CD28 and 4-1BB (3^rd^ gen)	IV	6.3 x 10^6^ to 2.6 x 10^10^	National Cancer Institute (NCI), USA	18	Completed (171); n=18 No DLT until highest dose which resulted in death 4 hours post infusion; 10/18 (55.6%) with grade 2 neurologic sx or seizure Median PFS = 1.3 months Median OS = 6.9 months
NCT06815029	Intracranial Genetically Modified Immune Cells (TGFβR2KO/IL13Rα2 CAR T-Cells) for the Treatment of Recurrent or Progressive Glioblastoma or Grade 3 or 4 IDH-Mutant Astrocytoma	ID: TGFβR2KO/IL13Rα2 CAR T-Cells scFv: anti- IL13Rα2 HR/TMD: NR CM: NR Armoring: TGFβR2 Genetic KO	IC	NR	City of Hope Medical Center, USA	27	Recruiting; No Results
NCT04903080	HER2-specific Chimeric Antigen Receptor (CAR) T Cells for Children with Ependymoma	ID: HER2 CAR T cells scFv: anti-HER2 HR/TMD: NR CM: NR	IV	DL-1: 5 x 10^7^/m^2^ DL1: 8 x 10^7^/m^2^	Pediatric Brain Tumor Consortium (multicenter), USA	50	Active, not recruiting; No Results
NCT04661384	Brain Tumor-Specific Immune Cells (IL13Ralpha2-CAR T Cells) for the Treatment of Leptomeningeal Glioblastoma, Ependymoma, or Medulloblastoma	ID: Autologous IL13(EQ)BBzeta/CD19t+ TCM-enriched T Cells scFv: anti-IL13Rα2 HR/TMD: NR CM: 4-1BB	ICV	NR	City of Hope Medical Center, USA	10	Active, not recruiting; No Results
NCT02442297	T Cells Expressing HER2-specific Chimeric Antigen Receptors (CAR) for Patients with HER2-Positive CNS Tumors	ID: HER2-specific T cells scFv: anti-HER2 HR/TMD: NR CM: NR	ICT	DL1: 1 x 10^7^ DL2: 3 x 10^7^ DL3: 10 x 10^7^ 3 Dosing Schedules	Baylor College of Medicine, USA	10	Active, not recruiting; No Results
CNS Lymphoma							
NCT07137494	A Study of MB-CART19.1 Cellular Therapy for People With Central Nervous System Lymphoma (CNSL)	ID: MB-CART19.1 scFv: anti-CD19 HR/TMD: CD8/CD8 CM: CD28	NR	NR	Memorial Sloan Kettering Cancer Center, USA	12	Recruiting; No Results
NCT07062627	Anbalcabtagene Autoleucel in Relapsed/Refractory CNS Lymphoma	ID: Anbal-cel scFv: anti-CD19 HR/TMD: CD8/CD8 CM: 4-1BB Armoring: Downregulation of PD-1 and TIGIT	IV	2 x 10^6^/kg to 2 x 10^8^/kg	Asan Medical Center, South Korea	12	Recruiting; No Results
NCT05651178	Human CD19-CD22 Targeted T Cells Injection for Refractory/Relapsed Central Nervous System Leukemia/Lymphoma Patients	ID: CD19-CD22 CAR-T scFv: anti-CD19-anti-CD22 (tandem) HR/TMD: NR CM: 4-1BB	IV and IT	For IV: DL1: 1 x 10^6^/kg DL2: 3 x 10^6^/kg DL3: 5 x 10^6^/kg For IT: DL1: 1 x 10^6^ cells	The Second Affiliated Hospital of Nanchang University, China	10	Recruiting; No Results
NCT05625594	Intracerebroventricular Administration of CD19-CAR T Cells (CD19CAR-CD28-CD3zeta-EGFRt-expressing Tn/Mem T-lymphocytes) for the Treatment of Central Nervous System Lymphoma	ID: CD19CAR-CD28-CD3zeta-EGFRt-expressing Tn/Mem T-lymphocytes scFv: anti-CD19 HR/TMD: NR CM: CD28	ICV	NR	City of Hope Medical Center, USA	20	Recruiting; No Results
NCT04608487	Axi-cel in CNS Lymphoma	ID: Yescarta^®^ scFv: anti-CD19 HR/TMD: CD28/CD28 CM: CD28	IV	2 x 10^6^/kg	Dana-Farber Cancer Institute, USA	18	Completed (206); n=18 CRS – 16/18 (89%); Grade 1-2 ICANS – 8/18 (44%); Grade 1-3 Median PFS = 14.3 months Median OS = 26.4 months
Pediatric Primary CNS Malignancies							
NCT07087002	GPC2-CAR T Cell Therapy for Relapsed or Refractory Medulloblastoma in Children and Young Adults	ID: GPC2-CAR T scFv: anti-GPC2 HR/TMD: NR CM: NR	ICV	NR	Lucile Packard Children’s Hospital Stanford, USA	18	Recruiting; No Results
NCT06221553	Safety and Efficacy of Loco-regional B7H3 IL-7Ra CAR T Cell in DIPG	ID: B7H3/IL-7Ra CAR T cell scFv: anti-B7H3 HR/TMD: NR CM: NR Armoring: IL7Ra gene cassette	IT	DL1: 1 x 10^7^ DL2: 3 x 10^7^ DL3: 10 x 10^7^	King Chulalongkorn Memorial Hospital, Thailand	9	Recruiting; No Results
NCT05835687	Loc3CAR: Locoregional Delivery of B7-H3-CAR T Cells for Pediatric Patients With Primary CNS Tumors	ID: B7-H3-CAR scFv: anti-B7H3 HR/TMD: CD8/CD8 CM: CD28 Addition: surface 4-1BBL expression	IT	1 x 10^7^ to 3 x 10^7^	St. Jude Children’s Research Hospital, USA	48	Recruiting; Interim results (207); n=8 AE: 8/8 (100%) with HA; 7/8 (87.5%) with fever n=7 available for survival analysis 2/9 (27%) achieved SD at week 8
NCT05768880	Study of B7-H3, EGFR806, HER2, And IL13-Zetakine (Quad) CAR T Cell Locoregional Immunotherapy For Pediatric Diffuse Intrinsic Pontine Glioma, Diffuse Midline Glioma, And Recurrent Or Refractory Central Nervous System Tumors	ID: SC-CAR4BRAIN scFv: anti-B7-H3-anti-EGFR806-anti-HER2-anti-IL13-zetakine (quad) HR/TMD: NR CM: NR	ICV	NR	Seattle Children’s Hospital, USA	72	Active, not recruiting; No Results
NCT05298995	GD2-CAR T Cells for Pediatric Brain Tumours	ID: iC9-GD2-CAR T-cells scFv: anti-GD2 HR/TMD: NR CM: 4-1BB and CD28 (3^rd^ gen) Safety switch: iCasp9	IV	1 x 10^6^/kg to 6 x 10^6^/kg	Bambino Gesù Hospital and Research Institute, Italy	54	Recruiting; No Results Preclinical publication (208)
NCT04510051	CAR T Cells After Lymphodepletion for the Treatment of IL13Rα2 Positive Recurrent or Refractory Brain Tumors in Children	ID: Autologous IL13(EQ)BBzeta/CD19t+ TCM-enriched T Cells scFv: anti-IL13Rα2 HR/TMD: IgG4/CD4 CM: 4-1BB	ICV	NR	City of Hope Medical Center, USA	18	Recruiting; Interim results (209); n=6 AE: 1/6 (16.7%) grade 3 HA, no grade 4 AE OM: n=5 available for survival analysis; 2/5 (40%) achieved SD; 3/5 (60%) with tumor shrinkage
NCT04196413	GD2 CAR T Cells in Diffuse Intrinsic Pontine Gliomas (DIPG) & Spinal Diffuse Midline Glioma (DMG)	ID: GD2CART scFv: anti-GD2 (14G2A) HR/TMD: CD8/CD8 CM: 4-1BB Safety Switch: iCasp9	IV or ICV	DL-1: 10 x 10^6^ DL1: 30 x 10^6^ DL2: 50 x 10^6^ ± 20%	Lucile Packard Children’s Hospital (LPCH), Stanford University, USA	97	Recruiting; Interim Results (210); n=4 P1 (DIPG) – Grade 1 CRS + TIAN; survived 3 months post CAR, 20 months post dx P2 (DIPG) – Grade 2 CRS + TIAN: survived 10 months post CAR, 26 months post dx P3 (DIPG) – Grade 1 CRS + TIAN; survived 7 months post CAR, 20 months post dx P4 (Spinal DMG) – Grade 3 CRS + ICANS/TIAN; survived 11 months post CAR, 2- months post dx
NCT04185038	Study of B7-H3-Specific CAR T Cell Locoregional Immunotherapy for Diffuse Intrinsic Pontine Glioma/Diffuse Midline Glioma and Recurrent or Refractory Pediatric Central Nervous System Tumors	ID: SCRI-CARB7H3(s) scFv: anti-B7H3 HR/TMD: NR CM: 4-1BB Safety: tEGFR tag	ICT or ICV	DL1: 1 x 10^7^ DL2: 2.5 x 10^7^ DL3: 5 x 10^7^ DL4: 10 x 10^7^	Seattle Children’s Hospital, USA	90	Recruiting; Interim Results (211); n=21 AE: 1/21 (4.8%) with DLT in DL2; 17/18 (81%) with HA and N/V; 13/21 (62%) with fatigue; 12/21 (57%) with fever; no CRS or ICANS OM: Median OS post CAR = 10.7 months; Median OS post-dx = 19.8 months OM: n=18 available for neuroimaging; 1/18 (6%) with PR; 15/18 (83%) with SD; 2/18 (11%) with PD
NCT04099797	C7R-GD2.CAR T Cells for Patients With GD2-expressing Brain Tumors (GAIL-B)	ID: C7R-GD2.CAR T cells scFv: anti-GD2 (14g2a) HR/TMD: CD8/CD8 CM: 4-1BB Armoring: IL7R gene cassette	IV and ICV	IV - DL1: 10 x 10^6^/kg ICV- DL1: 2 x 10^6^ DL2: 5 x 10^6^	Texas Children’s Hospital, Baylor University, USA	37	Recruiting; Interim Results (212, 213); n=11 n=8 received C7R-GD2.CAR T CRS – 6/8 (75%); Grade 1-4 TIAN – 7/8 (88%); Grade 1 7/8 (88%) eligible for repeat dosing OM: PR observed in 2/7 (29%) n=3 received GD2.CAR AE: No toxicity OM: PD after ≤ 3 weeks
NCT03638167	EGFR806-specific CAR T Cell Locoregional Immunotherapy for EGFR-positive Recurrent or Refractory Pediatric CNS Tumors	ID: EGFR806-specific chimeric antigen receptor (CAR) T cell scFv: anti-EGFR806 HR/TMD: NR CM: NR	ICT and ICV	1 x 10^7^ to 2.5 x 10^7^	Seattle Children’s Hospital, USA	11	Completed (214); n=4/11 (36.4%) treated AE: all ≤ grade 2; 1/4 (25%) with grade 1 seizure; 3/4 (75%) with grade 1–2 sensory changes, weakness, or urinary changes OM: 3/4 (75%) with PD; 1/4 (25%) with SD followed by CR to subsequent chemotherapy
NCT03500991	HER2-specific CAR T Cell Locoregional Immunotherapy for HER2-positive Recurrent/Refractory Pediatric CNS Tumors	ID: HER2-specific chimeric antigen receptor (CAR) T cell scFv: anti-HER2 HR/TMD: IgG4 (medium length) CM: 4-1BB Safety: tEGFR tag	ICT and ICV	DL1: 1 x 10^7^ DL2: 2.5 x 10^7^ DL3: 5 x 10^7^ DL4: 10 x 10^7^	Seattle Children’s Hospital, USA	10	Active, not recruiting; Interim Results (126); n=3 AE: 3/3 (100%) developed symptoms consistent with CRS (Grade 1-3) with fever, HA, and NSD most reported OM: at 4 weeks post infusion: 2/3 (66%) with PD; 1/3 (33%) with SD
CNS Metastases							
NCT06742593	Study of MT027 in Patients with Brain, Meninges, and Spinal Cord Metastatic Solid Tumors	ID: MT027 scFv: anti-B7H3 HR/TMD: NR CM: 4-1BB	IT	DL1: 1 x 10^7^ DL2: 1.5 x 10^7^ DL3: 2 x 10^7^ DL4: 2.5 x 10^7^	Cancer Hospital, Chinese Academy of Medical Sciences, China	12	Not yet recruiting; no results
NCT06592092	Clinical Study of QH104 Cell Injection for the Treatment of Meningeal Metastases of B7H3+ Solid Tumors	ID: Allogenic B7-H3 CAR-γδT cell (QH104) scFv: anti-B7-H3 HR/TMD: NR CM: NR	IT	DL1: 3 x 10^7^	Cancer Institute and Hospital, Chinese Academy of Medical Sciences, China	3	Completed (215); n=2 treated AE: none > grade 3; 1/2 (50%) with grade 1 absence seizure OM: At 30-day follow-up, 2/2 (100%) with SD
NCT03696030	HER2-CAR T Cells in Treating Patients With Recurrent Brain or Leptomeningeal Metastases	ID: HER2(EQ)BBzeta/CD19t+ T cells scFv: anti-HER2 HR/TMD: NR CM: 4-1BB Safety: CD19t tag	ICV	DL1: 22 x 10^6^ DL2: 110 x 10^6^ DL3: 220 x 10^6^	City of Hope Medical Center, USA	24	Active, not recruiting; Interim Results (216); n=23 AE: 2/23 (8.7%) experienced DLT with grade 3 HA OM: best result was SD with median duration of 56 days

+, estimated total reported via clinicaltrials.gov; NR, not reported; DL, dose level; ICT, intracavitary; ITm, intratumoral; IT, intrathecal; ICV, intracerebroventricular; IC, intracranial; SD, stable disease; cSD, confirmed stable disease (verified with f/u imaging); NSD, nervous system disorder; PD, progressive disease; DLT, dose-limiting toxicity; PR, partial response; CR, complete response; DCR, disease control rate; TVR, tumor volume reduction; N/V, nausea or vomiting; OM, outcome measure.

Both CRS and ICANS are systemic toxicity syndromes associated with CAR T-cell therapy. CRS results from the release of supraphysiologic concentrations of proinflammatory cytokines (i.e., IL-6, IL-1β IFN-γ, IL-10, TNFα, etc.) following engagement and activation of T-cells (infused or endogenous) or other immune effector cells (173). CRS typically initially presents as fever that evolves into hypotension, hypoxia, and end-organ damage (17, 174). CRS can progress to ICANS, which is characterized by a spectrum of cognitive dysfunction ranging from mild confusion, headache, and word-finding difficulty to severe encephalopathy, cerebral edema, seizures and coma; however, ICANS can also occur in the absence of CRS. Mechanistically, ICANS is not well understood, though it likely involves disruption of the blood brain barrier (BBB) through endothelial and glial cell damage, allowing an influx of proinflammatory cytokines into the CNS (175). Unlike CRS and ICANS whose etiology involves systemic inflammation, TIAN is a sequelae of local neuroinflammation. TIAN is divided into two non-mutually exclusive categories (i.e., Type 1 and Type 2) according to the mechanism of neurotoxicity (19, 176). Type 1 TIAN is characterized by a mass-effect-driven increase in intracranial pressure (ICP) via peri-tumoral accumulation of inflammatory vasogenic edema. The resulting mechanical stress can manifest as headache, vision changes, seizures, or in severe cases herniation and death. Type 2 TIAN is characterized by electrophysiologic neuronal dysfunction via dysregulated neuroimmune interactions, often manifesting as a worsening of baseline neurologic deficits. A validated grading system for TIAN severity was developed to mirror the grading systems of CRS and ICANS developed by the American Society of Transplantation and Cellular Therapy (ASTCT) (17). Both systems assess severity on a scale of 1 to 5, with 1 representing mild severity and 5 representing death attributable to the toxicity syndrome.

Several strategies are actively being explored to address CRS, ICANS, and TIAN resulting from CAR T-cell therapy for CNS malignancies. Proactive strategies to mitigate the risks (4)of CAR-related toxicity syndromes include locoregional administration to reduce systemic immune responses and dose optimization to prevent excessive cytokine production. Additionally, many of the fine-tuning CAR engineering strategies discussed above (i.e., tandem, dual/multi, synNotch, and logic-gated constructs) double as regulators of CAR activity, enabling the execution of precise activation programs following antigen recognition (177–179). While proactive strategies might reduce the baseline toxicity of CAR T-cell therapy, it is also important that unexpected toxic responses can be addressed after CARs have been administered. Currently, clinical management of CRS, ICANS, and TIAN involve conservative symptom management (IV fluids, Tylenol, O_2_) followed by the administration of tocilizumab (IL-6 receptor antagonist) and dexamethasone for CRS, dose escalation of dexamethasone and administration of anakinra (IL-1 receptor antagonist) for ICANS, and dexamethasone, administration of anakinra (IL-1 receptor antagonist), and CSF drainage depending on severity for TIAN (i.e., ICP > 20 mmHg CSF drainage may be considered) (19, 180, 181). Of note, although a TIAN scoring system has been developed (19), interventional protocols via expert consensus are less apparent. Appropriate identification of type 1 vs type 2 TIAN is important as clinical management may differ significantly (19). Type 1 requires careful monitoring as even mild cases can lead to life threatening complications and high-grade type I is considered a medical emergency. In contrast, type 2 management is often more conservative (i.e., symptom management) and pharmacological intervention is reserved for select cases (e.g., type II involving brainstem or cervical spine) (19). Novel management strategies, including both reversible and irreversible activity-modulating techniques are also under investigation. CAR activity can be reversibly inhibited with pharmacologic control of CAR signaling via dasatinib infusion and the integration of transcriptional regulatory systems (e.g., Tet-on and Tet-off systems) (182–184). Finally, to permanently eliminate CAR T-cells from circulation researchers have investigated the incorporation of inducible suicide genes (e.g., iCasp9) or integration of truncated (i.e., lacking intracellular signaling activity) CAR surface expressed receptors (e.g., tEGFR, tCD19, tCD34, etc.) for targeted elimination by monoclonal antibodies including cetuximab and rituximab (185–188).

### 3.3 CAR T-cell clinical trials against CNS malignancies

As of the writing of this paper, a total of 71 clinical trials investigating CAR T-cell therapy for primary and secondary CNS malignancies were reported on clinicaltrials.gov. Thirty-five (49%) exclusively targeted adult GBM, seventeen (24%) targeted adult GBM and other high-grade glioma (HGG), five (7%) targeted adult CNS lymphoma, eleven (15%) targeted pediatric GBM and HGG, and three (4%) targeted CNS metastases. Twenty-five (35%) of these clinical trials reported completed or interim results (n=278 patients receiving CAR T-cells).

In this section we will review these trials and provide a breakdown of outcome measures, CAR constructs, delivery and dosing data reported across all trials. We organize this discussion by disease entity, covering GBM in adult and pediatric patients, Mixed HGG and GBM, HGG and GBM in pediatric patients only, and finally CNS Lymphoma and metastatic disease. While many clinical trials report the CARs ARD, details regarding additional construct components such as the HR/TMD and CM are rarely published. Furthermore, not all trials report dose levels until data publication. Given the significant impact that construct composition has on the resulting immune response, persistence, and anti-tumor efficacy of CAR T-cells as well as the impact dosing and route of administration have on toxicity, we have reported all available construct details, dose levels and routes of administration associated with each trial in **Table 4**.

#### 3.3.1 Glioblastoma (adult and pediatric)

##### 3.3.1.1 Outcome measurements

For GBM trials enrolling both adults and pediatric patients, results have been published from five completed and seven in-progress trials, for a total of n=150 patients. Here we will discuss only completed trials that reported both 1. OS and 2. CAR-related adverse events (AEs) or clinical response with ≥ 10 patients, reflecting a total of three trials with n=101 patients.

In the largest of these clinical trials, Brown and colleagues at the City of Hope Medical Center conducted a phase I study (NCT02208362, n=65) in which 2.2 x 10^7^ to 2.2 x 10^8^ anti-IL13Rα2 CAR T-cells were administered via intracavitary (ICT), intracerebroventricular (ICV), or a combination in rGMB-majority HGGs. A total of n=58 patients received at least three doses and were evaluated for OS, AE, and clinical response. Superior median overall survival of 10.2 months was observed in the dual ICV/ICT arm, with a median OS of 8 months for all patients and a median OS of 7.7 months in the rGBM group (compared to the reported average median OS for rGBM of 4 to 11.8 months in patients receiving cytotoxic agents) (189). Additionally, AEs ≥ Grade 3 were observed in 40% (n=23) of patients, including encephalopathy (n=1, Grade 3), ataxia (n=1, Grade 3), and cerebral edema (n=2, Grade 4). With respect to clinical response, 50% (n=29) achieved stable disease (SD) or better and 22% (n=13) achieved imaging-confirmed SD or better (131).

O’Rourke and colleagues at the University of Pennsylvania published interim results from a phase I study (NCT02209376, n=10) investigating the intravenous administration of anti-EGFRvIII CARs in adult patients with GBM. Patients received 1.75 x 10^8^ to 5.0 x 10^8^ anti-EGFRvIII total CARs. The authors report a median OS of 251 days (~8 months) with one patient exceeding 450 days (14.8 months) on trial. Furthermore, a total of nine AEs ≥ grade 3 observed in three patients were reported (172).

Ahmed and colleagues at Baylor College of Medicine published survival, AE, and clinical response results from a phase I trial (NCT01109095, n=16) in patients with GBM receiving 1.0 x 10^7^ to 1.0 x 10^8^ anti-HER2 CAR T-cells intravenously. A total (adult and pediatric patients) median OS of 22.4 months and an adult median OS of 30 months from diagnosis. No serious AE or CRS were observed. With respect to clinical response, 50% (n=8) demonstrated objective response: one patient had partial response with a ~62% reduction in tumor volume lasting 8 months and seven patients experienced stable disease for more than 6 weeks (of these 5 were durable >10 weeks). At the time of this trial’s publication, three patients continued follow-up 24 to >30 months following infusion of CAR T-cells (190).

Together, these trials demonstrate that ≥ 100 million CAR T-cells can be safely administered either locoregionally, intravenously, or a combination of the two in the treatment of GBM in mixed adult/pediatric populations. Furthermore, these trials contribute valuable data points supporting the potential to achieve superior survival outcomes (median OS up to 30 months) compared to standard therapies (median OS of 12.3 months, see **Table 2**). Despite the promise of this modality, the risk of CAR related AEs remains a considerable concern with AE ≥ Grade 3 reported in 26 patients (~26% of all patients reported). Nevertheless, the potential of this therapeutic modality to significantly improve clinical outcomes in one of the most devastating diseases with historically dismal prognoses is nothing short of revolutionary. In the following section, the constructs, dosing and delivery routes of all thirty-five trials noted in **Table 4** will be briefly discussed.

##### 3.3.1.2 CAR constructs, delivery and dosing

Of the thirty-five GBM clinical trials reviewed, thirty-three (94%) reported the scFv, six (17%) reported the HR/TMD, and seventeen (49%) reported the CM used in their CAR construct. Additionally, seven unique routes of administration with weight-based doses ranging from one- to one-hundred million per kg and total CAR doses ranging from one- to nine-hundred million CAR T-cells were investigated. Below we provide a breakdown of reported CAR constructs, delivery, and dosing.

scFv: Reported in 33 trials with 15 unique antigens. Most to least commonly employed: anti-EGFRvIII used in nine trials, anti-B7-H3 used in six trials, anti*-m*EGFR-*hIL13R*α*2* used in three trials, anti-GD2 used in two trials, chlorotoxin used in two trials, and anti-IL13Rα2 used in two trials. The remaining antigen targets, anti-B7H3/IL13Rα2, anti-DLL3, anti-GPC3, anti-CD44/CD133, anti-CD70, anti-NKG2D, anti-CD147, anti-PDL1, and anti-HER2 were all used in one trial. scFvs specific to completed trials include anti-EGFRvIII (3/5), anti-IL13Rα2 (1/5), and anti-HER2 (1/5). HR/TMD: Reported in 6 trials with 2 unique domains. CD8 is used in four trials and CD28 is used in two trials. Of the completed trials, CD8 is used in (2/5) with the remaining trials not reporting. CM: Reported in 17 trials with 3 unique domains. 4-1BB used in ten trials, CD28 used in five trials, and combination of 4-1BB and CD28 (3^rd^ generation) used in two trials. CM specific to complete trials include 4-1BB (3/5), CD28 (1/5), and a combination of 4-1BB and CD28 (1/5). Delivery and Dosing: Combination ICV and IV (dose NR), combination ICV and ITm (5 x 10^6^ to 9 x 10^8^), ICV only (2.5 x 10^6^ to 2.5 x 10^8^), IV only (4.5 x 10^6^/kg to 1.5 x 10^8^/kg or 1 x 10^6^ to 5 x 10^8^ or 1 x 10^6^/m^2^ to 1.0 x 10^8^/m^2^), ICT (2.2 x 10^7^ to 2.2 x 10^8^), ITm (1 x 10^7^ to 5 x 10^7^), and IT (5 x 10^6^ to 6 x 10^7^). 3/5 completed trials administered IV (4.5 x 10^6^/kg to 1.5 x 10^8^/kg or 1 x 10^6^ to 5 x 10^8^ or 1 x 10^6^/m^2^ to 1.0 x 10^8^/m^2^), 1/5 administered ICT, ICV, or ICT/ICV dual delivery (2.2 x 10^7^ to 2.2 x 10^8^), and 1/5 not reported.

#### 3.3.2 Mixed high-grade glioma and glioblastoma (adult only)

##### 3.3.2.1 Outcome measurements

For clinical trials specifically investigating mixed populations of GBM and other primary HGG, results have been published from one completed trial and two in-progress, for a total of n=31 patients. All trials will be discussed below, regardless of what outcome measures were reported given the sparsity of patient data available.

Goff and colleagues with the National Cancer Institute published the results of a completed dose-escalating phase I clinical trial (NCT01454596, n=18) investigating the intravenous administration of third-generation anti-EGFRvIII CAR T-cells in patients with recurrent GBMs. Patients received doses ranging from 6.3 x 10^6^ to 2.6 x 10^10^ CAR T-cells. The authors reported that, as expected, all patients developed grade 3 or 4 cytopenias due to lymphodepleting chemotherapy, though no DLT was observed until reaching the highest concentration of infused cells (6 x 10^10^ total cells: 3.41 x 10^10^ non-transduced + 2.59 x 10^10^ transduced, CAR+ T-cells). This dose ultimately resulted in treatment related death (TRD, Grade 5 dyspnea/hypoxia) with significant pulmonary edema noted on postmortem analysis. Median OS was 6.9 months (IQR: 2.8 to 10 months) with three patients surviving at least 1 year and one surviving >59 months. Serial MRI imaging revealed no objective response in any of the patients, with median PFS of 1.3 months.

Gwak and colleagues with the National Cancer Center in the Republic of Korea published interim results from a phase I trial (NCT05540873, n=10) employing anti-IL13Rα2 CAR T-cells in patients with Grade 3–4 glioma. Patients received an intravenous infusion of 1 x 10^7^/kg to 1 x 10^8^/kg CAR T-cells. No DLT was observed and all AE were ≤ grade 3, with the most common being pyrexia (70%) and elevated transaminases (70%). Nine patients were eligible for survival analysis, with best response being stable disease in three patients. Median PFS was 2.60 months (95% confidence interval (CI), 0.19-5.01) and median OS was 10.9 months (95% CI, 7.9-14.3).

Lin and colleagues with Capital Medical University in Beijing published interim results from a phase I trial (NCT03423992, n=3) employing anti-EphA2 CAR T-cells in patients with GBM. Patients received an intravenous dose of 1 x 10^6^/kg CAR T-cells. Two patients (67%) experienced grade 2 CRS with no other AE reported. One patient responded with SD and two patients with PD, with overall survival ranging from 86 to 181 days.

While all three trials together only represent thirty-one total patients, they nevertheless contribute to the growing body of evidence of CAR T-cell safety and efficacy in primary adult CNS malignancies. For example, Goff and colleagues with the NCI demonstrated a clear upper limit of cell product that can be safely administered intravenously. They also highlighted an under-reported aspect of CAR T-cell therapy by specifically addressing the total number of T-cells infused, not just the number of CAR^+^ T-cells. Failure to disclose such information introduces reproducibility constraints and limits the generalizability of the findings.

##### 3.3.2.2 CAR constructs, delivery and dosing

All seventeen trials reported the scFv used, two reported the HR/TMD, and five reported the CM. Five unique routes of administration with weight-based doses ranging from one million to one-hundred million per kg and total CAR doses ranging from one million to twenty-six billion CARs were employed. Below we provide a breakdown of reported CAR constructs, delivery, and dosing.

scFv: Reported in 17 trials where one trial used 2 CARs and one trial used 6 CARs for total of 23 CARs. 11 unique antigens were employed. Most to least common: anti-IL13Rα2 used in five trials, anti-HER2 used in four trials, anti-B7-H3 used in three trials, anti-EGFRvIII used in two trials, anti-CD70 used in two trials, anti-GD2 used in two trials. The remaining antigens, anti-CD133, anti-EphA2, anti-GARP, anti-EGFR806, anti-MUC1 were all used in one trial. HR/TMD: 2 reported; both were CD8/CD8; CM: 5 reported with 2 unique domains employed. 4-1BB only was used in four trials while CD28 + 4-1BB (3^rd^ gen) was used in one trial. Delivery and Dosing: Combination ITm and ICT (dose NR), ICT (6 x 10^7^ to 2.5 x 10^8^), IV (1 x 10^6^/kg to 1 x 10^8^/kg or 6.3 x 10^6^ to 2.6 x 10^10^), IT (1 x 10^6^/kg), ICV (6 x 10^7^ to 2.5 x 10^8^), and intracranial (IC, dose NR).

#### 3.3.3 Mixed high-grade glioma and glioblastoma (pediatric only)

##### 3.3.3.1 Outcome measurements

For clinical trials specifically investigating CAR T-cell use in pediatric (children and young adult) patients, results have been published from one completed and six in-progress clinical trials, for a total n=57 patients. All seven trials will be discussed below given the sparsity of patient data available.

Vitanza and colleagues with Seattle Children’s Hospital reported interim results from a phase 1 clinical trial (NCT03500991, “BrainChild01”, n=3) investigating the locoregional infusion of anti-HER2 CAR T-cells in children and young adults. Patients received repeated ICV and ICT doses of 1 x 10^7^ to 10 x 10^7^ CAR T-cells. No DLT were observed. The authors report both clinical as well as correlative laboratory evidence of local CNS immune activation. All three patients developed symptoms consistent with Grade 1–3 CRS. At 4 weeks post infusion two patients (66%) exhibited progressive disease (PD) and one patient (33%) exhibited SD.

Gust and colleagues with Seattle Children’s Hospital published the results of a completed phase I clinical trial (NCT03638167, “BrainChild02”, n=4) investigating the locoregional delivery of anti-EGFR806 CAR T-cells in pediatric patients (age 7 to 17 reported) with primary CNS malignancies (n=3 with HGG, n=1 with atypical teratoid rhabdoid tumor). Patients received weekly intracranial infusions of 1 x 10^7^ to 2.5 x 10^7^ CAR T cells into the tumor resection bed or the lateral ventricle via an implanted catheter over the course of 5 to 10 weeks. All treatment-related AE were ≤ grade 2, with headache being the most common. Three patients (75%) experienced progressive disease, and one patient (25%) had stable disease followed by full response to subsequent chemotherapy. Of the patients with PD response, two were available for survival analysis with OS of 12 and 19 months following initiation of CAR T-cell therapy. The patient with SD response eventually relapsed and died 2 years after receiving CAR T-cells.

Vitanza and colleagues with Seattle Children’s Hospital published interim results from a phase I clinical trial (NCT04185038, “BrainChild03”, n=21) investigating the locoregional administration of anti-B7-H3 CAR T-cells in pediatric patients with DIPG. Patients received repeated doses ranging from 1 x 10^7^ up to 10 x 10^7^ B7-H3 CAR T-cells using intra-patient dose-escalation regimens without previous lymphodepletion. A single case of DLT occurred as grade 4 intracranial hemorrhage in a 3-year-old patient. The median OS of all treated patients from their initial CAR T-cell infusion was 10.7 months (range: 0.6–45.8 months) and the median time from diagnosis to death (or last contact for survivors) was 19.8 months with 3 patients still alive 44.6, 45.6 and 52.5 months from diagnosis. A total of n=18 patients received enough CAR T-cells to have clinical response assessed via MRI, with 1 partial response (PR; 6%), 15 stable disease (SD; 83%) and 2 PD (11%).

Bertrand and colleagues with St. Jude published interim results from a phase I clinical trial (NCT05835687, “Loc3CAR”, n=8) investigating the locoregional delivery of anti-B7-H3 CAR T-cells in pediatric patients with r/r primary CNS malignancy (cohort 1) or post-radiation DIPG (cohort 2). Patients received six ICV infusions of 1 x 10^7^ to 3 x 10^7^ CARs over the course of 7 weeks. A single case of DLT occurred in a patient with large tumor burden and high B7-H3 expression (H-score = 300) with unresponsiveness and decorticate posturing that improved with CSF removal. Two of seven (29%) evaluable patients achieved clinical benefit with stable disease at week 8.

Wang and colleagues with City of Hope published interim results from a phase I clinical trial (NCT04510051, n=6) investigating the locoregional administration of anti-IL13Rα2 CAR T-cells in pediatric patients with CNS tumors. Patients received weekly intraventricular infusions, with the first infusion at a dose of 10 x 10^6^ CAR^+^ T-cells and subsequent infusions at 50 x 10^6^ CAR^+^ T-cells. No DLT was observed, however one patient did drop out after developing grade 3 catheter-related infection and was not evaluable for disease response. Overall, two of five evaluable patients achieved stable disease, and three of five evaluable patients had some tumor shrinkage on MRI, though all responses were transient.

Majzner and colleagues with Stanford University published interim results from a phase I clinical trial (NCT04196413, n=4) investigating the intravenous administration of anti-GD2 CAR T-cells in pediatric patients with H3K27M-mutated DIPG (n=3) or spinal cord DMG (n=1). Patients received 1 x 10^6^/kg CAR T-cells intravenously, with those demonstrating clinical benefit receiving subsequent CARs intracerebroventricularly. Patient 1 (DIPG) experienced Grade 1 CRS + TIAN with an OS of 3 months post CAR and 20 months post diagnosis. Patient 2 (DIPG) experienced Grade 2 CRS and TIAN with an OS of 10 months post CAR and 26 months post diagnosis. Patient 3 (DIPG) experienced Grade 1 CRS and TIAN with an OS of 7 months post CAR and 20 months post diagnosis. Patient 4 (Spinal DMG) experienced Grade 3 CRS and ICANS/TIAN with an OS of 11 months post CAR and 20 months post diagnosis.

Lin and colleagues with Texas Children’s Hospital published interim results from a phase I clinical trial (NCT04099797, n=11) investigating the intravenous administration of IL-7R-augmented (cohort 1, n=8) and non-augmented (cohort 2, n=3) anti-GD2 CAR T-cells in pediatric patients with high-grade CNS tumors. Patients in cohort 1 received GD2.CARTs alone (1 x 10^7^ cells/m^2^), and those in cohort 2 received C7R-GD2.CARTs at two dose levels (1 x 10^7^ cells/m^2^; 3 x 10^7^ cells/m^2^). There were no DLT reported in either cohort, no AE reported in cohort 1, grade 1 TIAN reported in seven of eight patients (88%) in cohort 2, grade 1 CRS in five of eight (63%) patients in cohort 2 and grade 4 CRS in one of eight patients (13%) in cohort 2. All eight patients in cohort 2 experienced temporary improvement from baseline neurologic deficits (range of 2 to >12 weeks) and seven of eight (88%) remained eligible for additional treatment cycles (range 2–4 cycles).

These trials demonstrate that up to one-hundred million CAR T-cells can be safely administered systemically or locoregionally in pediatric patients with high-grade CNS malignancies. Compared to standard therapies for diffuse midline glioma with median OS of 12 and 17.3 months, respectively, CAR T-cells demonstrate the potential to extend median OS by up to 50% (achieving median OS of 18 to 26 months), though clinical outcomes remain dismal. Of note, ~50% of reported data arise from a single institution (Seattle Children’s Hospital) which may limit the generalizability of the current evidence. Thus, continued efforts to expand upon the knowledge that these trials have generated is essential in the fight against pediatric CNS malignancies.

##### 3.3.3.2 CAR constructs, delivery and dosing

All eleven trials reported the scFv used, five reported the HR/TMD used, and seven reported the CM used. CARs were delivered using four unique routes of administration with weight-based doses ranging from one million to six million per kg and total CAR doses ranging from ten million to one-hundred million CAR T-cells. Below we provide a breakdown of reported CAR constructs, delivery, and dosing.

scFv: 11 reported; Most to least commonly employed: anti-B7H3 and anti-GD2 used in three trials, anti-GPC2, anti-B7-H3-anti-EGFR806-anti-HER2-anti-IL13-zetakine (quad), anti-IL13Rα2, anti-EGFR806, and anti-HER2 all used in one trial. HR/TMD: 5 reported with three unique domains. CD8/CD8 used in three trials, IgG4/CD4 and IgG4 (medium length) used in one trial. CM: 7 reported with three unique domains. 4-1BB used in five trials, CD28, and 4-1BB + CD28 (3^rd^ generation) used in one trial, respectively. Delivery and Dosing: Combination ICV and ICT (1 x 10^7^ to 1 x 10^8^), ICV (1 x 10^7^ to 1 x 10^8^), IT (1 x 10^7^ to 1 x 10^8^), and IV (1 x 10^6^/kg to 6 x 10^6^/kg).

#### 3.3.4 CNS lymphoma and metastatic disease

##### 3.3.4.1 Outcome measurements

While CNS lymphoma and CNS metastasis are distinct oncological entities, they are reported together due to the vastly limited data available for each. One completed trial investigating CNSL has been published. One completed and one in-progress trial for CNS metastasis have been published. Together, a total of n=43 patients have been reported on for CAR T-cells in CNS lymphoma and CNS metastasis. All three trials will be discussed below given the sparsity of patient data available.

Chukwueke and colleagues with Dana-Farber Cancer Institute published the results of a completed pilot study (NCT04608487, n=18) investigating the intravenous administration of anti-CD19 CAR T-cells (Axi-cel) for primary and secondary CNS lymphoma. Patients received 2 x 10^6^/kg CAR T-cells following lymphodepletion with fludarabine and cyclophosphamide. Sixteen (89%) patients developed CRS ≤ grade 3 and eight (44%) developed ICANS, five (28%) of which were ≥ grade 3. The median PFS was 14.3 months (95% CI: 6.3-NR) and median OS was 26.4 months (95% CI: 11.2-NR).

Wang and colleagues with the Chinese Academy of Medical Sciences published the results of a completed phase 1 clinical trial (NCT06592092, n=2) investigating the intrathecal infusion of allogeneic B7H3-targeted CAR-γδT cells in patients with leptomeningeal metastasis of lung adenocarcinoma. Patients received a single dose of 3 x 10^7^ cells via lumbar puncture or Ommaya reservoir infusion. No AE ≥ Grade 3 were observed; however, one patient did experience an absence seizure one day after infusion. Both patients demonstrated SD at 30 days post-infusion with a reduction or complete elimination of tumor cells within the CSF. Furthermore, both patients experienced clinical improvement of LM symptoms.

Portnow and colleagues with City of Hope Medical Center published interim results of a phase 1 clinical trial (NCT03696030, n=23) investigating the locoregional administration of anti-HER2 CAR T-cells in breast cancer patients with recurrent brain and/or leptomeningeal metastases with and without lymphodepletion. During the DLT evaluation phase, patients received three weekly doses of either 2.2 x 10^7^, 1.1 x 10^8^, or 2.2 x 10^8^ CAR T-cells. The first ten patients did not receive lymphodepletion while the subsequent eight did. DLT occurred in two patients due to Grade 3 headaches. The best response achieved in both groups was stable disease (SD). In the CAR T only group, 44% achieved SD with a median duration of 56 days (range 50–136 days). In the LD+CAR T group, 64% achieved SD with a median duration of 56 days (range 44–134 days). Though limited by low patient enrollment and the outcome measures reported, these trials highlight the potential of CAR T-cells to improve the clinical outcomes of CNS lymphoma and metastases.

##### 3.3.4.2 CAR constructs, delivery and dosing

All eight trials reported the scFv used, three reported the TMD/HR used, and seven reported the CM used. Three unique routes of administration were used with weight-based doses ranging from two million to two-hundred million per kg and total CAR doses ranging from one million to two-hundred twenty million CAR T-cells. Below we provide a breakdown of reported CAR constructs, delivery, and dosing.

scFv: 8 reported; most to least commonly employed: anti-CD19 used in four trials, anti-B7H3 used in two trials, anti-CD19-anti-CD22 (tandem), and anti-HER2 used in one trial. HR/TMD: 3 reported with 2 unique domains. CD8 is used in two trials and CD28 is used in one trial. CM: 7 reported with 2 unique domains. CD28 is used in three trials and 4-1BB is used in four trials. Delivery and Dosing: IV (2 x 10^6^/kg to 2 x 10^8^/kg), IT (1 x 10^6^ cells to 3 x 10^7^), and ICV (2.2 x 10^7^ to 2.2 x 10^8^).

### 3.4 Discussion and future directions

In this review, we have discussed the history of the chimeric antigen receptor and how it has evolved into most common CAR designs employed in both preclinical and clinical settings today. We highlight several fundamental knowledge gaps with respect to the molecular identity and function of structural CAR domains that if explored further might allow us to exploit natural immunological processes to improve anti-tumor efficacy and safety. Additionally, we provide an overview of both primary and secondary CNS malignancies, noting key epidemiological data points regarding incidence and survival. Regarding the current landscape of CAR T-cells in CNS malignancies, we address key challenges preventing wide-spread clinical adoption of this therapy as well as several promising strategies, both ongoing and theoretical, to address these challenges.

A total of seventy-one clinical trials were reviewed (up to date as of March 2026) using the following search parameters: “CAR T-cell” for Intervention/Treatment and “CNS Neoplasm”, “CNS Tumor”, “CNS Malignancy”, “Brain Tumor”, “Brain Cancer”, “Glioma”, Glioblastoma”, CNS Lymphoma” and “CNS Metastasis” for condition/disease. We followed-up our investigation of these trials with targeted searches for publications associated with each clinical trial ID. Using this strategy, we were able to identify results from several completed trials, as well as interim results from ongoing trials and preliminary results from pre-clinical studies. We also included data regarding method of delivery, dosages used, the location of the study, and all available information regarding the construct(s) used during each trial. As the clinical utility of CAR T-cells continue to expand, it is imperative that the current strategies being explored to advance the technology promote universal growth across the entire field.

A wide net (i.e., exploration of a vast diversity of molecular construct designs, delivery methods and dosages) has been cast to maximize the breadth of data that can be collected regarding CAR T-cells for CNS malignancies. While useful as a means of rapidly growing the field, this comes at a trade-off with granular clarity of understanding/applying clinically beneficial constructs, dosing, and routes of administration. This is an outstanding area of research that will need to be addressed via preclinical testing. A specific focus on understanding and refining the best possible construct for the disease of interest rather than quickly applying one that works will likely lead to greater clinical success. In addition, requiring reporting on all aspects of CAR constructs used in clinical trials may help to identify previously unappreciated interactions between certain domains (i.e., scFV-HR, HR-TM, TM-CM, etc.). Additional areas of research need are determining the necessity, method, and consequences of lymphodepletion for CAR T-cell therapy in CNS malignancies.

Of equal importance is the balance of trying to expeditiously test preclinical successes in the clinical environment. As with all fields, clinical studies lag preclinical data by years. There are current clinical studies still actively recruiting/investigating that are based on successfully tested and verified constructs preclinically from almost a decade ago. This is further illustrated by the fact that preclinical studies and recent clinical studies in hematology have demonstrated that generation of CAR T-cells from naïve or memory-like phenotype T-cells results in superior expansion, persistence, and anti-tumor efficacy *in vivo (*191, 192). However, few studies employing CAR T-cells for CNS malignancies have tested this clinically, as currently most are still positively selecting for more broad linage markers (i.e., CD4, CD8). This concept may be overly idealistic as there are clear administrative, regulatory, and financial barriers to running clinical trials.

Meaningful advances in CAR T-cell therapy will likely come from thoughtful strategies that employ novel approaches while still considering the balance between efficacy and toxicity. There are many promising candidates that span the breath of CAR T-cell design and application. These include unique targets (i.e., scFV), bispecific and bicstronic CARs, original culture methods (i.e., skewing CARs toward memory phenotypes), novel combination strategies, and, of particular interest to our lab, administration strategies (e.g., chronotherapy). Incorporating our knowledge and applying it to future clinical trials and other cellular therapies (i.e., *in vivo* CAR T or CAR NK) will hopefully lead to significant scientific advancement and meaningful benefit to patients.
